# Asprosin Aggravates Tubular Epithelial Cell Injury and Phenotypic Transformation via Mitochondrial Dynamics Disorder Mediated by Excessive Drp1 SUMOylation in Diabetic Nephropathy Mice

**DOI:** 10.1002/advs.202503259

**Published:** 2025-08-27

**Authors:** Qianqian Huang, Xiaowei Xiong, Sheng Chen, Yuan Wang, Li Wang, Wentao Liu, Chen Liu, Guohua Zeng, Qiren Huang

**Affiliations:** ^1^ Jiangxi Provincial Key Laboratory of Drug Target Discovery and Validation School of Pharmacy Jiangxi Medical College Nanchang University Nanchang Jiangxi 330006 China; ^2^ Department of Cardiovascular Surgery The First Affiliated Hospital Jiangxi Medical College Nanchang University Nanchang Jiangxi 330006 China

**Keywords:** asprosin, diabetic kidney disease, mitochondrial dynamics, SUMOylation

## Abstract

Epidemiological studies show that some diabetic patients develop end‐stage renal dysfunction without significant proteinuria or glomerulopathy, underscoring the role of renal tubular epithelial cell (TEC) impairment in diabetic kidney disease (DKD). However, the primary pathogenic determinants underlying TEC impairment and disease advancement in DKD progression remain unclear. This study reveals that asprosin (ASP) is up‐regulated and positively correlated with kidney dysfunction in DKD mice. Moreover, elevated ASP is mainly located in the renal TEC, and negatively impacts TEC. In addition, supraphysiological ASP concentration impairs mitochondrial dynamics and function in both DKD mice and HK2 cells. Mechanistically, ASP promotes Drp1 over‐SUMOylation, thus reducing Drp1 degradation and disrupting mitochondrial dynamics homeostasis. However, the mutation of Drp1‐SUMOylation modification sites alleviates the mitochondrial dynamics disorder, TEC injury, and phenotypic transformation induced by ASP. Also, it is further elucidated that such a regulatory effect of ASP on the Drp1‐SUMOylation modification is fulfilled by modulating PIAS1 or SENP1 (a de‐SUMOylation protease). Importantly, either adipose tissue‐specific ASP deficiency (ASP^−/−^) or ASP antibody (AASP) intervention significantly alleviates the kidney injury and mitochondrial dynamics disorder induced by STZ/HFD. These findings identify ASP as a novel predictor of DKD and offer new therapeutic strategies for DKD prevention and treatment.

## Graphical abstract

ASP aggravates TEC injury and phenotypic transformation via mitochondrial dynamics disorder mediated by excessive Drp1 SUMOylation in DKD mice. However, either ASP deficiency or AASP effectively alleviates the kidney injury and mitochondrial dynamics disorder induced by STZ/HFD, highlighting that ASP could be an early diagnostic marker and potential therapeutic target of DKD.

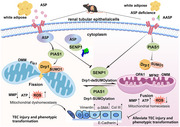

## 1 Introduction

Diabetic kidney disease (DKD) is one of the major microvascular complications of diabetic patients and has become a major cause of end‐stage renal disease (ESRD) worldwide.^[^ ^1^, ^2^ ^]^ Renal tubular epithelial cells (TEC) are the main sites of glucose reabsorption, and their metabolic modes change with environmental changes.^[^ ^3^ ^]^ For example, under the glycotoxic condition, the metabolic mode of TEC switches from mitochondrial oxidative metabolism to glycolysis, which promotes the proliferation and differentiation of TEC.^[^ ^4^ ^]^ The process known as TEC epithelial‐mesenchymal transition (EMT), marked by epithelial cell polarity disruption and intercellular adhesion, along with the acquisition of mesenchymal characteristics, is widely acknowledged as a significant factor in the progression of DKD.^[^ ^5^ ^]^ It is worth noting that TEC phenotypic transformation plays a central role in the progression of renal tubulointerstitial fibrosis (RTF) and is an important pathological basis for ESRD. However, the current clinical diagnosis and treatment rationales have not been able to effectively prevent the development of ESRD.^[^ ^6^ ^]^ Therefore, it is urgent to ascertain the etiology and mechanism of DKD pathogenesis.

It is commonly thought that re‐absorption is the predominant function of TEC, especially proximal tubules, and the process of active re‐absorption requires a large amount of energy. Thus, the proximal tubule is enriched with a large number of mitochondria due to its high energy demand and dependence on aerobic metabolism.^[^ ^7^, ^8^ ^]^ Mitochondria are dynamic organelles that change to acclimate to the environment for survival and growth, including continuous fission and fusion.^[^ ^9^ ^]^ Clinical data show that mitochondrial fragmentation is increased in renal cortex tubular cells in the early stage of DKD patients.^[^ ^10^ ^]^ Disruptions of mitochondrial dynamics directly impact mitochondrial function, including proper biogenesis, autophagy, and redox state etc.^[^ ^11^ ^]^

It is well‐established that mitochondrial dynamics homeostasis is essential for maintaining the normal function of TEC. Among the numerous lines of evidence, dynamin‐related protein 1 (Drp1), a fission protein, plays a central role in altered dynamics, and its expression is increased in TEC of DKD mice. A key step in Drp1‐mediated mitochondrial fission is the translocation of Drp1 from the cytoplasm to the mitochondrial outer membrane (MOM).^[^ ^12^ ^]^ It is reported that post‐translational modification of Drp1 is involved in its translocation to MOM.^[^ ^13^ ^]^ Some studies show that Drp1 SUMOylation promotes the association of Drp1 with MOM, and reduces its lysosomal degradation, thus further enhancing the mitochondrial fission.^[^ ^14^, ^15^ ^]^ Therefore, inhibiting the excessive modification of Drp1 by SUMO1 might be an important approach for maintaining the mitochondrial dynamics homeostasis and function in the progression of DKD.

SUMOylation is a highly dynamic cascade that is catalyzed by the sequential enzymes, including E1 (SAE1/2), E2 (Ubc9), and E3 ligase in a manner analogous to ubiquitination, and it is also reversed by SUMO‐specific proteases, which cover six isoenzymes, including SENP1‐3 and SENP5‐7.^[^ ^12^ ^]^ Protein inhibitors of activated STAT (PIAS) family proteins were originally identified as inhibitors of STAT, and they also possess E3 SUMO ligase property.^[^ ^16^ ^]^ Our previous study has shown that PIAS1 is involved in vascular endothelial insulin resistance induced by glucose and lipid metabolism disorders.^[^ ^17^ ^]^ SENP1, as a SUMO‐specific protease, plays an important regulatory role in adipogenesis,^[^ ^18^ ^]^ glucose and lipid metabolism.^[^ ^19^, ^20^ ^]^ These existing data suggest that PIAS1 and SENP1 are involved in regulating the development of multiple metabolic diseases. However, it is unclear whether PIAS1 and SENP1 are involved in Drp1‐SUMO1 modification and regulate mitochondrial dynamics in the development of DKD.

Asprosin (ASP), a peptide hormone mainly from white adipose tissue, was discovered in 2016 linked to a genetic mutation causing neonatal progeroid syndrome. Later studies show it affects appetite, metabolism, and heart health. High levels of ASP are linked to unhealthy habits, obesity, diabetes, and other metabolic disorders. Animal studies suggest that blocking ASP could improve metabolism, making it a potential treatment target.^[^ ^21^, ^22^, ^23^ ^]^ Our recent studies have demonstrated that ASP inhibits white adipose browning,^[^ ^24^ ^]^ promotes vascular endothelial insulin resistance,^[^ ^25^ ^]^ vascular endothelial inflammation,^[^ ^26^ ^]^ and aggravates metabolism‐associated fatty liver disease,^[^ ^27^ ^]^ suggesting that ASP is closely associated with the development of metabolic disorders. Several studies have shown that a supraphysiological concentration of ASP is an independent risk factor for the development and progression of DKD.^[^ ^28^, ^29^, ^30^ ^]^ However, the role of ASP in DKD progression remains largely unexplored. It has not been reported yet whether ASP is involved in renal tubular injury or by what mechanisms it works. Resolving the above issues is of great significance for early marker discovery and DKD therapy.

In the present study, we indicate a positive correlation between ASP levels and renal function related indicators in DKD. In addition, we demonstrate that the regulation of the DKD process by ASP is realized primarily through promoting the excessive modification of Drp‐SUMO1, which leads to the disturbance of mitochondrial dynamics. In this regulation process, SENP1 and PIAS1 play important roles and would also be the potential targets. Altogether, these findings suggest that ASP could be an early diagnostic marker and a potential therapeutic target of DKD.

## 2 Results

### 2.1 ASP Intervention Causes or Exacerbates TEC Injury and Phenotypic Transformation

**Figure 1:**
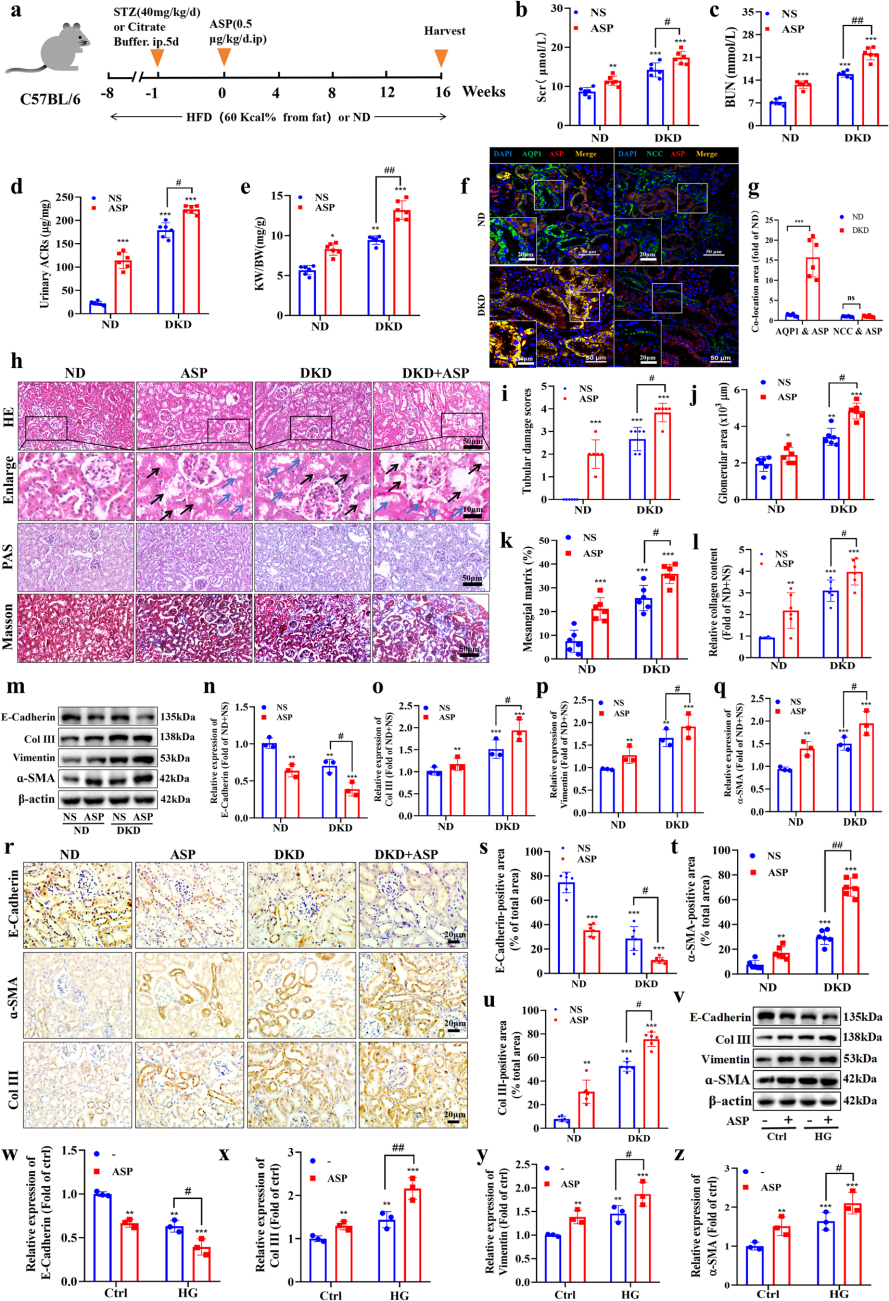
ASP intervention causes or exacerbates TEC injury and phenotypic transformation. a) Schematic of the experimental design. Normal or STZ/HFD‐induced T2DM mice were treated with either normal saline (NS) or 0.5 µg· kg^−1^· d^−1^ ASP for 16 weeks. b,c) The levels of Scr and BUN (n = 6). d,e) The urinary albumin to creatinine ratios (ACRs) and kidney weight (KW)/body weight (BW) ratio in mice (n = 6). f,g) Co‐localization of ASP with different segment‐specific tubular markers of DKD kidney. ASP (red), aquaporin 1 (AQP1, marker of proximal renal tubular epithelial cell, green), Na^+^‐Cl^−^ cotransporter (NCC, marker of distal renal tubular epithelial cell, green), DAPI (blue). Scale bar: original images, 50 µm; enlarged images, 20 µm. h–l) Representative images and quantitative analysis of (h, i, j) hematoxylin and eosin (H&E, black arrow: swelling, blue arrow: vacuolation), (h, k) periodic acid–Schiff (PAS) staining, and (h, l) Masson's trichrome staining of kidneys (n = 6). Scale bar: original images, 50 µm; enlarged images, 10 µm. m–q) Representative Western blotting and quantitative data showing the expressions of E‐Cadherin, Collagen III (Col III), Vimentin and α‐SMA of kidneys in various treatment groups. β‐actin was used as loading controls (n = 6). r–u) Representative images of immunohistochemical staining and quantitative data of (r, s) E‐Cadherin, (r, t) α‐SMA and (r, u) Collagen III (Col III) of kidneys after various treatment(n = 6). Scale bar: 20 µm. v–z) Representative Western blotting and quantitative data showing the expressions of E‐Cadherin, Collagen III (Col III), Vimentin and α‐SMA of HK2 cells under various treatment conditions. β‐actin was used as loading controls (n = 3). Data were presented as mean ± SEM. Statistical analysis was performed using the two‐way ANOVA followed by Tukey's multiple comparison test in all panels except for (a), (f), (h), (i), (m), (r) and (v). Non‐parametric Mann‐Whitney test was applied for statistical analysis of tubular injury scores (i). All tests were two tailed. ^*^ *P*<0.05, ^**^ *P*<0.01, ^***^ *P*<0.001 versus ND+NS or Ctrl; ^#^ *P*<0.05, ^##^ *P*<0.01, ^###^ *P*<0.001 versus DKD+NS or HG.

To investigate the link between ASP and DKD, we detected the serum ASP levels in DKD mice. We found that the levels of serum ASP were significantly increased starting with week 4 in DKD mice (*P*<0.001), and there was a gradually increased trend with the progression of DKD (Figure S1a, Supporting Information); in addition, the levels of urinary ASP in DKD mice were much higher than those of ND mice (Figure S1b, Supporting Information). Moreover, the ASP levels were positively correlated with the levels of serum creatinine (Scr) (Figure S1c, Supporting Information) and blood urea nitrogen (BUN) (Figure S1d, Supporting Information). To further investigate the role of ASP in DKD pathogenesis, ASP intervention was applied to the T2DM mice induced by STZ/HFD (**Figure** **1**a). Our data showed that under the ND‐feeding, ASP intervention significantly elevated the Scr and BUN levels (Figure 1b,c), urinary ACRs levels (Figure 1d), and kidney weight/body weight ratio (KW/BW) (Figure 1e). Moreover, under the DKD, ASP intervention aggravated the elevations of the above parameters, suggesting that ASP intervention causes and worsens the DKD induced by STZ/HFD. Considering that ASP can regulate systemic glycolipid metabolism, we next observed the effects of ASP on glycolipid metabolism in mice. Our results revealed that ASP intervention caused and aggravated the glycolipid metabolism disorder and elevated systolic blood pressure (SBP) in DKD mice (Figure S2a–m, Supporting Information), suggesting that the pro‐DKD effect of ASP may be due to in part inducing the glycolipid metabolism disorder.

The renal tubule is the main site of glucose re‐absorption and it is the most vulnerable when it is subjected to high glucose stress. In order to further explore the direct impact and mechanism of ASP on the progression of DKD, we examined the ASP in situ expression in different segments of renal tubules by immunofluorescence. The data displayed that ASP was co‐located with proximal tubule marker aquaporin 1 (AQPI), but not with distal tubule marker Na^+^‐Cl^−^ co‐transporter (NCC), indicating that ASP is mainly located in proximal renal tubule epithelial cells (Figure 1f,g) and it may play a significant role in the function of proximal tubules in DKD mice.

We examined the impacts of ASP on TEC in DKD mice. Our morphological data showed that ASP caused TEC swelling (black arrow), vacuolation (blue arrow, H&E, Figure 1h,i), glomerular hypertrophy (H&E, Figure 1h,j), mesangial matrix expansion (PAS, Figure 1h,k) and collagen deposition (Masson, Figure 1h,l). Western blotting (Figure 1m–q) and immunohistochemistry (Figure 1r–u) revealed that ASP treatment resulted in and even exacerbated the TEC phenotypic transformation induced by DKD, evidenced as lower expression of E‐Cadherin and higher expression of Col III, Vimentin, and α‐SMA. Similar results were obtained from in vitro experiments (Figure 1v–z). Also, our in vitro data revealed that the promoting phenotypic transformation effect of ASP was concentration‐dependent (Figure S3a–e, Supporting Information). Besides, the wound‐healing assay showed that ASP promoted or further enhanced the migration of HK2 cells induced by HG (Figure S4a,b, Supporting Information). Altogether, these data indicate that ASP intervention causes and even exacerbates the TEC injury and phenotypic transformation both in vivo and in vitro.

### 2.2 ASP Deficiency Inhibits TEC Injury and Phenotypic Transformation

**Figure 2:**
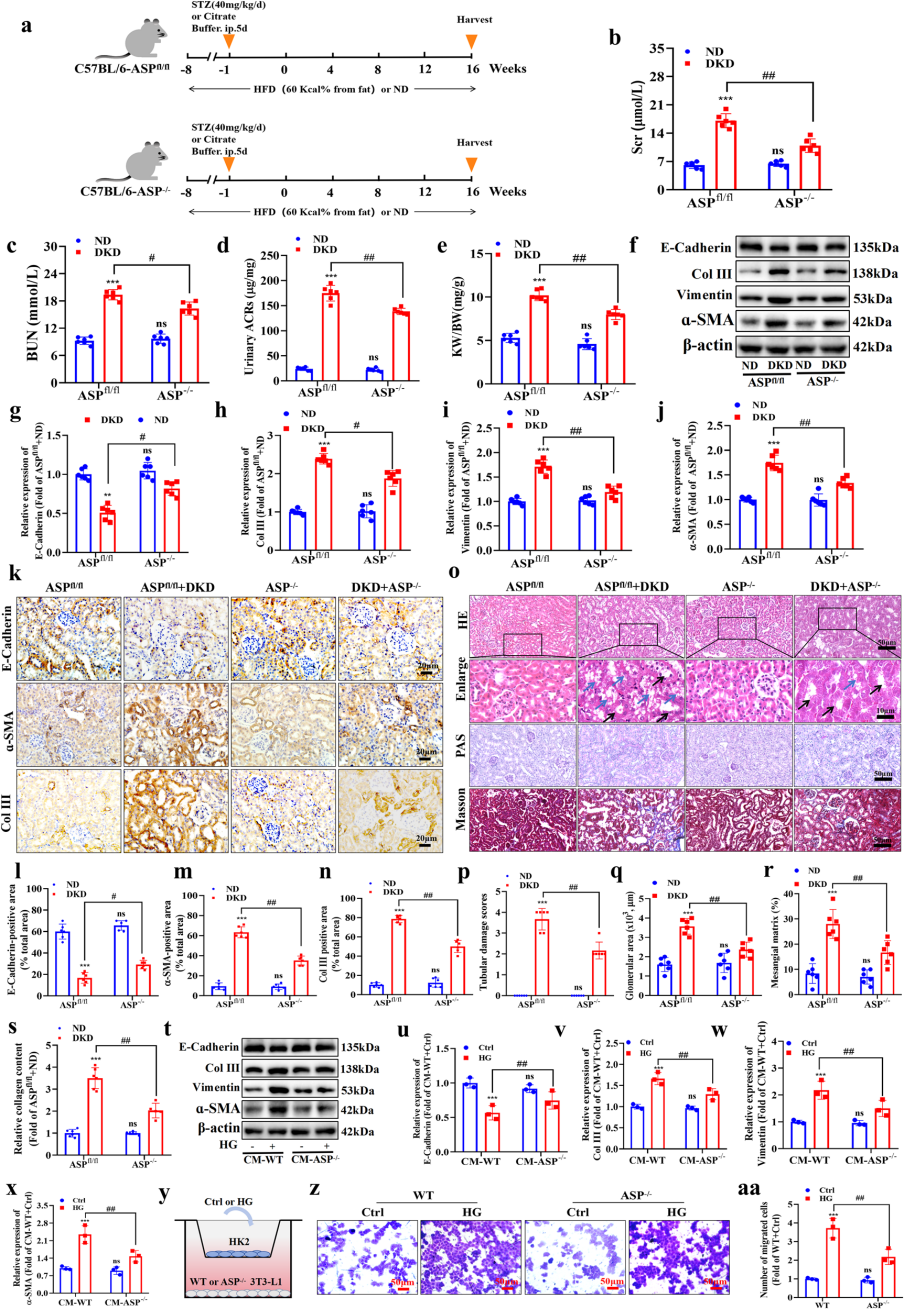
ASP deficiency inhibits TEC injury and phenotypic transformation. a) Schematic of the experimental design. b, c) The levels of Scr and BUN in mice (n = 6). d, e) The urinary ACRs and kidney weight (KW)/body weight (BW) ratio in mice (n = 6). f–j) Representative Western blotting and quantitative data showing the expressions of E‐Cadherin, Collagen III (Col III), Vimentin and α‐SMA in kidneys of ASP^fl/fl^ and ASP^−/−^ mice. β‐actin was used as a loading control (n = 6). k–n) Representative images of immunohistochemical staining and quantitative data of (k, l) E‐Cadherin, (k, m) α‐SMA, and (k, n) Collagen III (Col III) in kidneys of ASP^fl/fl^ and ASP^−/−^ mice (n = 6). Scale bar: 20 µm. o‐s) Representative images and quantitative data of (o–q) hematoxylin and eosin (H&E, black arrow: swelling, blue arrow: vacuolation), (o, r) periodic acid–Schiff (PAS) staining, and (o, s) Masson's trichrome staining and quantitative of collagen content of kidneys (n = 6). Scale bar: original images, 50 µm; enlarged images, 10 µm. t–x) Representative Western blotting and quantitative data showing the expressions of E‐Cadherin, Collagen III (Col III), Vimentin, and α‐SMA of HK2 cells. β‐actin was used as a loading control (n = 3). y) The co‐culture system of HK‐2 cells and 3T3‐L1 cells. z and aa) Representative images and quantitative data of the Transwell experiment of HK2 cells (n = 3). Scale bar: 50 µm. Data were presented as mean ± SEM. Statistical analysis was performed using the two‐way ANOVA followed by Tukey's multiple comparison test in all panels except for (a), (f), (k), (o), (p), (t), (y), and (z). The nonparametric Mann‐Whitney test was applied for statistical analysis of tubular injury scores (p). All tests were two tailed. ^**^ *P*<0.01, ^***^ *P*<0.001 versus ASP^fl/fl^+ND or CM‐WT+Ctrl; ^#^ *P*<0.05, ^##^ *P*<0.01 versus ASP^fl/fl^+DKD or CM‐WT+HG. ns, no significant difference versus ASP^fl/fl^+ND or CM‐WT+Ctrl.

To further explore the role of ASP in DKD progression, an adipose tissue‐specific ASP‐deficient (ASP^−/−^) mouse model was established (Figure S5a–e, Supporting Information). Next, to evaluate the impact of ASP^−/−^ on the development of DKD, ASP^fl/fl^ and ASP^−/−^ mice were treated as **Figure** **2**a. The results exhibited that ASP^−/−^ significantly alleviated the increased levels of Scr (Figure 2b), BUN (Figure 2c), urinary ACRs (Figure 2d), and KW/BW (Figure 2e) induced by DKD. In addition, ASP deficiency alleviated glucose and lipid metabolic disorder in DKD mice (Figure S6a–m, Supporting Information), which suggests that ASP^−/−^ protects TEC in DKD mice by improving glucose and lipid metabolic disorder. Western blotting showed that E‐Cadherin was increased and Col III, Vimentin, and α‐SMA were decreased in ASP^−/−^+DKD group compared with the DKD group (Figure 2f–j). Immunohistochemistry analysis also showed similar results (Figure 2k–n). The results of pathological staining showed that, the degree of renal tubular injury in ASP^−/−^+ DKD group was reduced compared with DKD group, including the reductions of TEC swelling (black arrow), vacuolation (blue arrow, H&E, Figure 2o,p), glomerular hypertrophy (H&E, Figure 2o,q), mesangial matrix expansion (PAS, Figure 2o,r) and collagen deposition (Masson, Figure 2o,s). Besides, we detected serum FFA and adipokines, including leptin and adiponectin in DKD mice. The results showed that the disorders of adipokines occurred in DKD mice, and ASP deficiency significantly relieved the disorders in DKD mice (Figure S7a–c, Supporting Information). These results suggest that ASP^−/−^ reverses the damage and phenotypic transition of TEC in the progression of DKD.

To mimic the environment of adipose tissue‐specific ASP^−/−^ in vivo, HK2 cells were treated with the conditioned media (CM) from either WT‐3T3‐L1 adipocytes or ASP^−/−^‐3T3‐L1 adipocytes. Western blotting data showed that the treatment with CM‐ASP^−/−^ markedly reversed the phenotypic transition induced by HG in HK2 cells, characterized as increased E‐Cadherin (Figure 2t,u), and decreased Col III (Figure 2t,v), Vimentin (Figure 2t,w) and ɑ‐SMA (Figure 2t,x). Another character is the enhancement of migration. To better ascertain the effects of ASP^−/−^ on migration of HK2 cells in vitro, a Transwell co‐culture system (0.8 µm) of 3T3‐L1 adipocytes and HK2 cells was established (Figure 2y). The results showed that ASP deficiency markedly reversed the elevated migration induced by HG (Figure 2z and aa). Taken together, these data suggest that ASP^−/−^ inhibits the DKD progression both in vivo and in vitro.

### 2.3 ASP Intervention Causes or Aggravates Mitochondrial Dysfunction by Disturbing Mitochondrial Dynamics Homeostasis

To further investigate the mechanism by which ASP promotes the progression of DKD, we performed the transcriptome analysis of the kidneys in ASP^fl/fl^+DKD and ASP^−/−^+DKD mice. KEGG pathway enrichment analysis showed that the differential expression genes (DEGs) were significantly enriched in the oxidative phosphorylation (OXPHOS), thermogenesis, citrate cycle (TCA cycle) pathway (**Figure** **3**a), which were associated with mitochondrial metabolism and function. The heatmap showed that compared with the DKD mice, the gene expressions responsible for mitochondrial OXPHOS, thermogenesis, and TCA cycle were markedly elevated in the ASP^−/−^+DKD mice (Figure 3b), suggesting that ASP^−/−^ promotes glycolipid metabolism by enhancing mitochondrial function in DKD mice. Mitochondrial dynamics homeostasis is indispensable for the maintenance of its function. Next, we evaluated the effects of ASP intervention on the mitochondrial dynamics of TEC. Western blotting data revealed that ASP intervention caused or exacerbated the mitochondrial dynamics disturbance in DKD mice, manifested as elevated Drp1 and Fis1 levels as well as decreased OPA1 and MFN2 levels in mitochondria (Figure 3c–e). Numerous studies have shown that Drp1 plays a central role in mitochondrial fission.^[^ ^31^, ^32^ ^]^ Therefore, here we focused primarily on the regulatory effects of ASP on Drp1. Immunofluorescence assay showed that ASP intervention promoted the co‐localization of Drp1 and Tomm20 (outer mitochondrial membrane marker), and the combined intervention of ASP and STZ/HFD further enhanced the levels of co‐localization (Figure 3f,g), indicating that ASP promotes Drp1 translocation from the cytosol to the mitochondria. In addition, we found that ASP increased the phosphorylation levels of Drp1 at s616 (Figure S8a,b, Supporting Information). Moreover, ASP intervention significantly reduced ATP levels (Figure 3h) and increased production of mitochondrial ROS (Figure 3i,j). These data suggest that ASP results in mitochondrial dysfunction by disrupting the mitochondrial dynamics homeostasis in DKD mice.

Figure 3ASP intervention causes or aggravates mitochondrial dysfunction by disturbing mitochondrial dynamics homeostasis. a) Analysis of KEGG enrichment pathways for differentially expressed genes (DEGs) in the kidneys of DKD and DKD+ASP^−/−^ mice (n = 3). b) Heatmap of DEGs related to mitochondrial function in the kidneys of ASP^fl/fl^+DKD and ASP^−/−^+DKD mice (n = 3). c–e) Representative Western blotting and quantitative data showing the expressions of OPA1, MFN2, Drp1, and Fis1 in the cytosolic fraction and mitochondrial fraction of renal tissue. β‐actin was used as a marker for the cytoplasmic fraction, and COX IV was used as a marker for the mitochondrial fraction (n = 6). f, g) Co‐localization and its analysis of Drp1 with Tomm20 in kidneys (n = 6). Drp1 (red), Tomm20 (green), DAPI (blue). Scale bar: original images, 50 µm; enlarged images, 20 µm. h) ATP levels in kidneys (n = 6). i, j) Representative images of MitoSOX (red) and its quantitative data in kidneys (n = 6). Scale bar: 50 µm. k, l) Representative images of MitoTraker (red) and its quantitative data in HK2 cells (n = 3). Scale bar: original images, 10 µm; enlarged images, 5 µm. m, n) Representative images of transmission electron microscope (TEM) and quantitative data in HK2 cells (n = 3). Scale bar: original images, 500 nm; enlarged images, 200 nm. o–q) Representative Western blotting and quantitative data showing the expressions of OPA1, MFN2, Drp1, and Fis1 in the cytosolic fraction and mitochondrial fraction of HK2 cells. β‐actin and COX IV were used as loading controls (n = 3). r, s) Co‐localization of Drp1 with MitoTraker and quantitative data in HK2 cells (n = 3). MitoTraker (red), Drp1 (green), DAPI (blue). Scale bar: original images, 20 µm; enlarged images, 5 µm. t) ATP levels in HK2 cells (n = 3). u, v) Detection and quantification of Mito‐SOX in HK2 by Flow cytometry. w, x) Representative images of JC‐1 staining and its quantitative data in HK2 cells (n = 3). Scale bar: 50 µm. Data were presented as mean ± SEM. Statistical analysis was performed using one‐way (d, e, p, q, v) or two‐way ANOVA (g, h, j, l, n, s, t, x) followed by Tukey's multiple comparison test. All tests were two tailed. ^*^ *P*<0.05, ^**^ *P*<0.01, ^***^ *P*<0.001 versus ND+NS or Ctrl; ^#^ *P*<0.05, ^##^ *P*<0.01, ^###^ *P*<0.01 versus DKD or HG.

**Figure:**
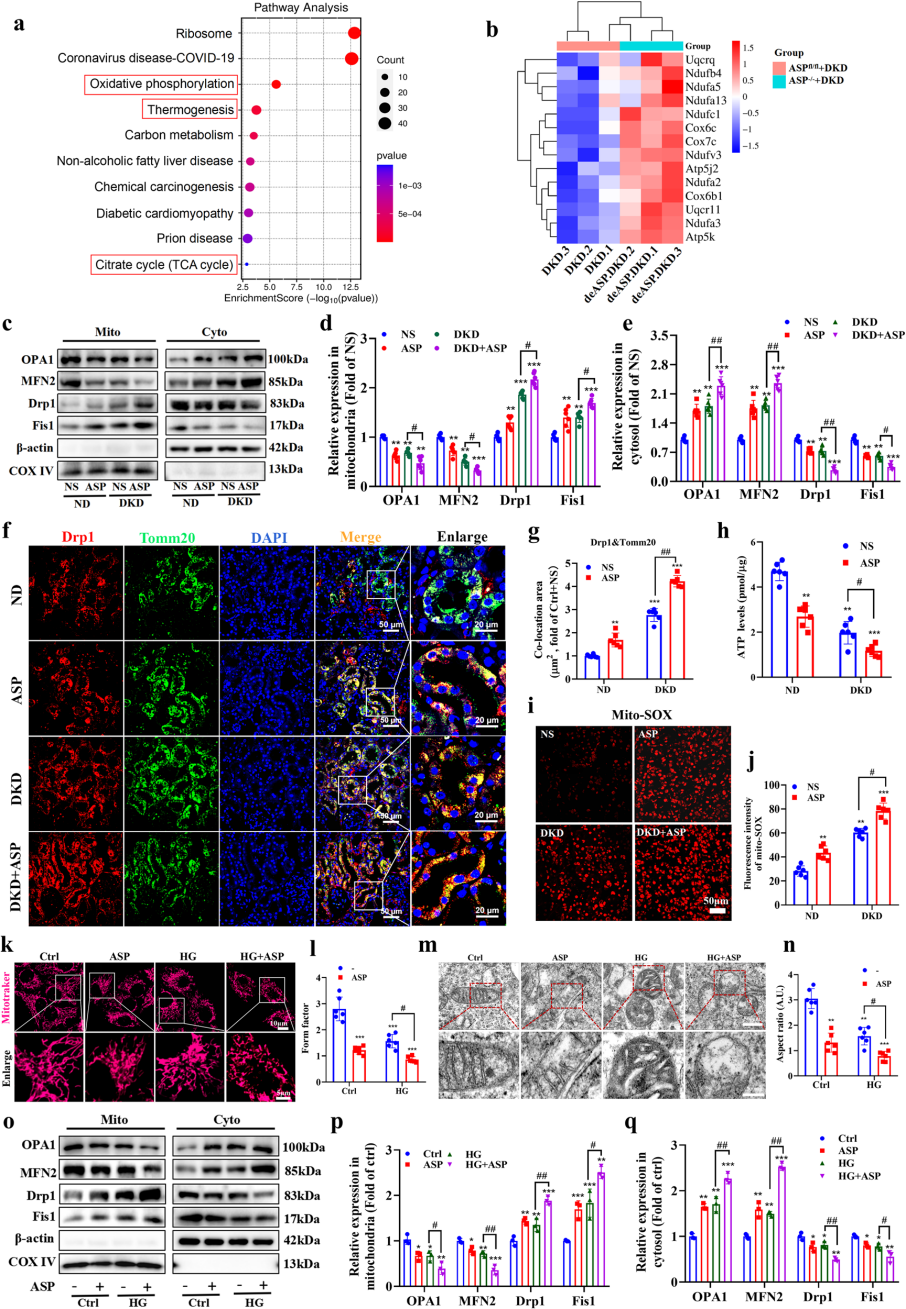

**Figure:**
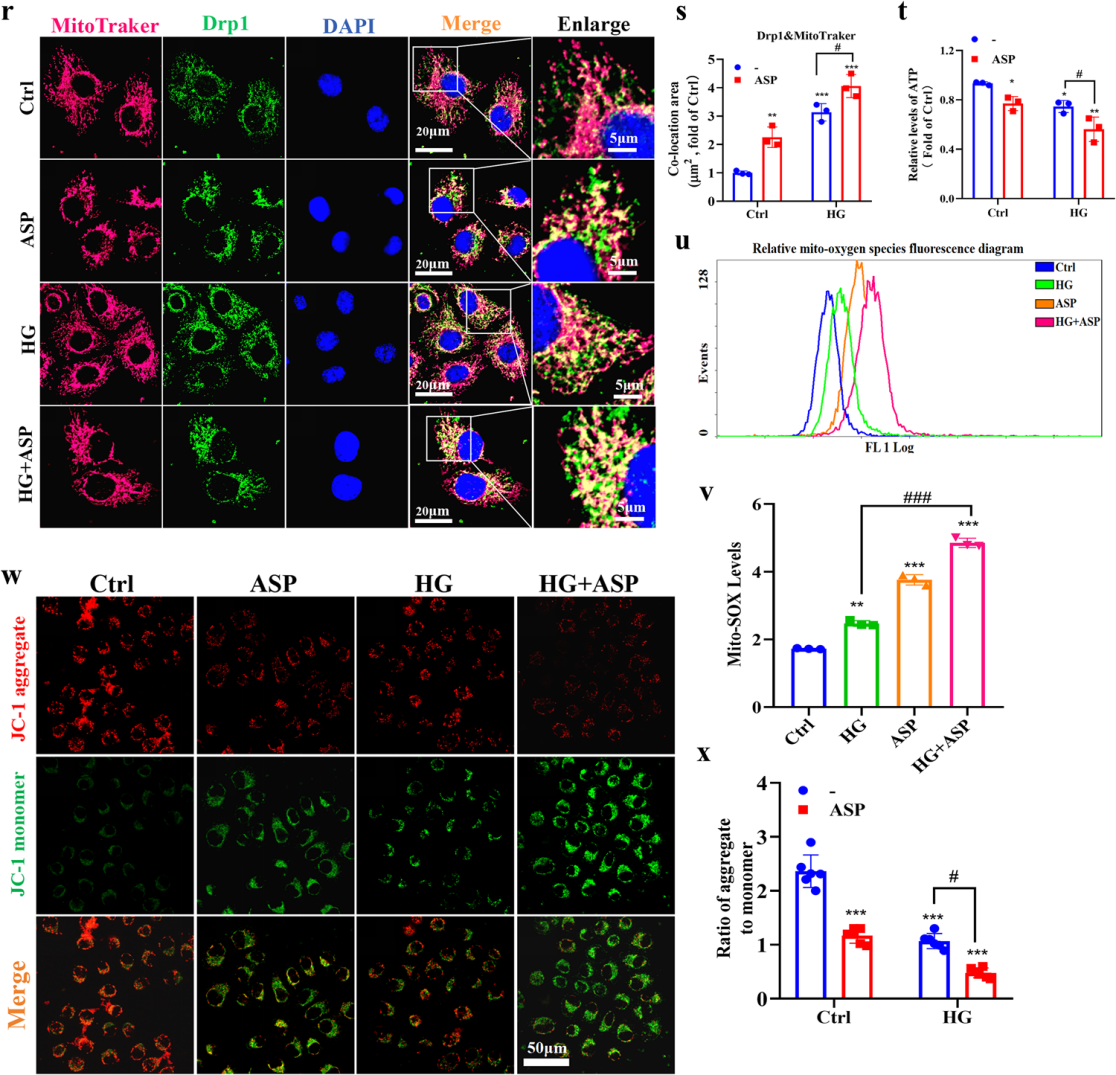

Subsequently, Mito‐Tracker and transmission electron microscope (TEM) were used to examine the morphology of mitochondria in HK2 cells. Our results showed that ASP promoted or exacerbated mitochondrial fragmentation induced by HG (Figure 3k–n). Moreover, the expressions of mitochondrial dynamics related proteins were similar to those in vivo experiments (Figure 3o–q). Also, the results of immunofluorescence showed that ASP promoted the Drp1 expression induced by HG in mitochondria (Figure 3r,s). These results suggest that ASP may promote mitochondrial fission in HK2 cells. Meanwhile, ASP decreased ATP production (Figure 3t), increased mitochondrial ROS levels (Figure 3u,v), and promoted the conversion of JC‐1 polymers to monomers (mitochondrial membrane potential decreased, Figure 3w,x), demonstrating that ASP intervention alone impairs mitochondrial function, and ASP+HG intervention further aggravates the mitochondrial dysfunction. These data suggest that ASP results in mitochondrial dysfunction by disrupting the mitochondrial dynamics homeostasis in HK2 cells.

### 2.4 ASP Deficiency Alleviates Mitochondrial Dysfunction by Restoring Mitochondrial Dynamics Homeostasis

**Figure 4:**
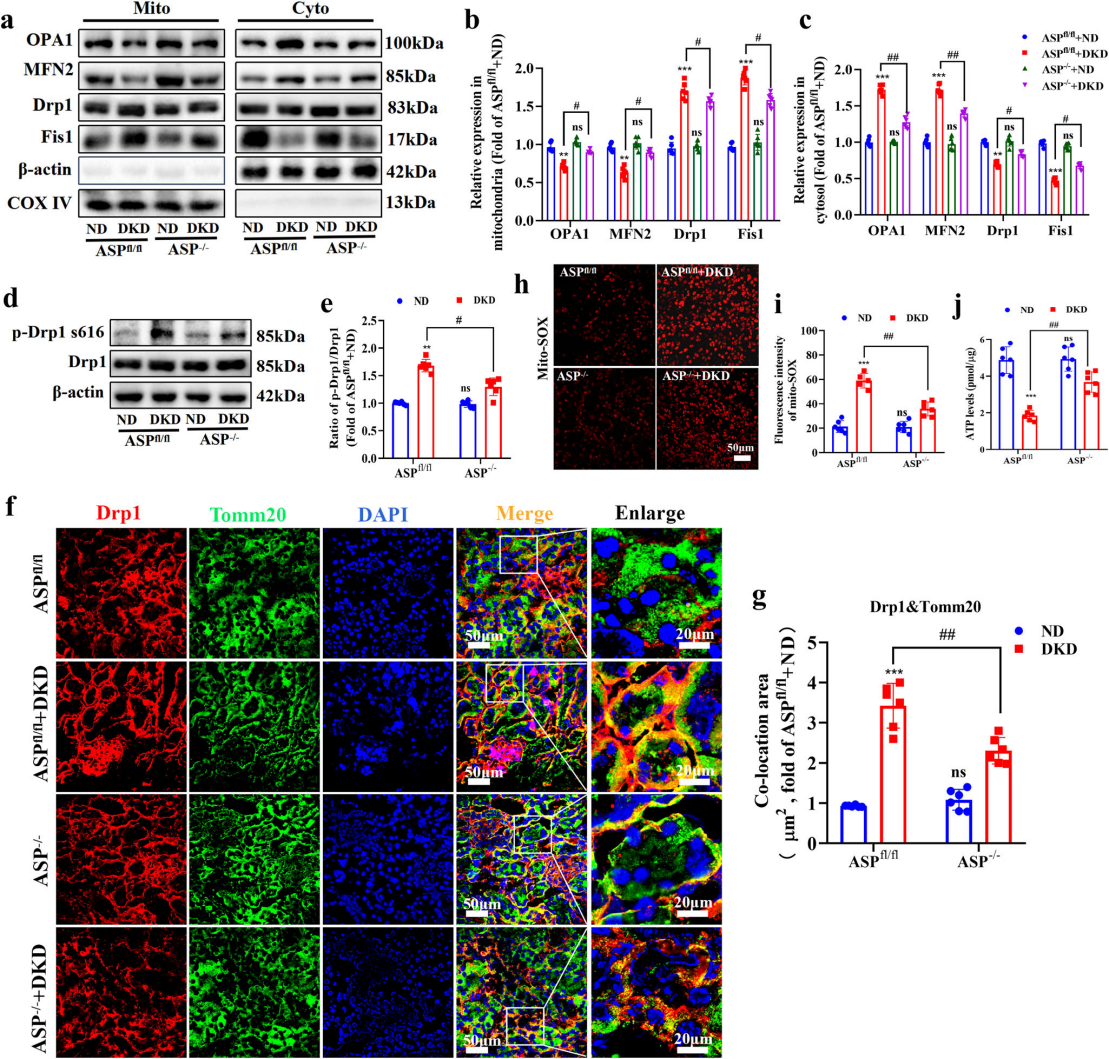
ASP deficiency alleviates mitochondrial dysfunction by restoring mitochondrial dynamics homeostasis. a–c) Representative Western blotting and quantitative data showing the expressions of OPA1, MFN2, Drp1, and Fis1 in cytosolic fraction and mitochondrial fraction in kidneys. β‐actin was used as a marker for the cytoplasmic fraction, and COX IV was used as a marker for the mitochondrial fraction (n = 6). d, e) Representative Western blotting and quantitative data showing the expressions of p‐Drp1 s616 and Drp1 in kidneys. β‐actin were used as loading controls (n = 6). f, g) Co‐localization of Drp1 with Tomm20 and its quantitative data in kidneys (n = 6). Drp1 (red), Tomm20 (green), DAPI (blue). Scale bar: original images, 50 µm; enlarged images, 20 µm. h, i) Representative images of MitoSOX (red) and its quantitative data in kidneys (n = 6). Scale bar: 50 µm. j) ATP levels in kidneys (n = 6). Data were presented as mean ± SEM. Statistical analysis was performed using one‐way (b, c) or two‐way ANOVA (e, i, j, g) followed by Tukey's multiple comparison test. All tests were two tailed. ^**^ *P* < 0.01, ^***^ *P* < 0.001 versus ASP^fl/fl^+ND; ^#^ *P* < 0.05, ^##^ *P* < 0.01 versus ASP^fl/fl^+DKD. ns, no significant difference versus ASP^fl/fl^+ND.

We have found that ASP intervention impaired mitochondrial dynamics and function in the kidneys of DKD mice. Subsequently, we explored the effects of ASP deficiency on mitochondrial dynamics and function. The results showed that compared with DKD group, the expressions of mitochondrial fission proteins Drp1 and Fis1 in mitochondria were decreased, while the expressions of fusion proteins OPA1 and MFN2 in mitochondria were increased in the kidneys of ASP^−/−^+DKD mice (**Figure** **4**a–c). To further explore the effects of ASP^−/−^ on Drp1, Western blotting and immunofluorescence co‐localization assay were performed. We found that ASP^−/−^ significantly reduced the expression levels of p‐Drp1 s616 in the kidneys of DKD mice (Figure 4d,e), and decreased the co‐localization of Drp1 and Tomm20 (Figure 4f,g). These data suggest that ASP^−/−^ alleviates the mitochondrial dynamics disorder in DKD mice. At the same time, we detected the indicators related to mitochondrial function, and the results revealed that ASP^−/−^ significantly reduced mitochondrial ROS levels (Figure 4h,i) and increased ATP levels (Figure 4j) in the kidneys of DKD mice. These results indicate that ASP^−/−^ has protective effects on mitochondrial function in the kidneys of DKD mice.

### 2.5 Pro‐Injury and Phenotypic Transformation Effects of ASP are Mediated by Drp1

**Figure 5:**
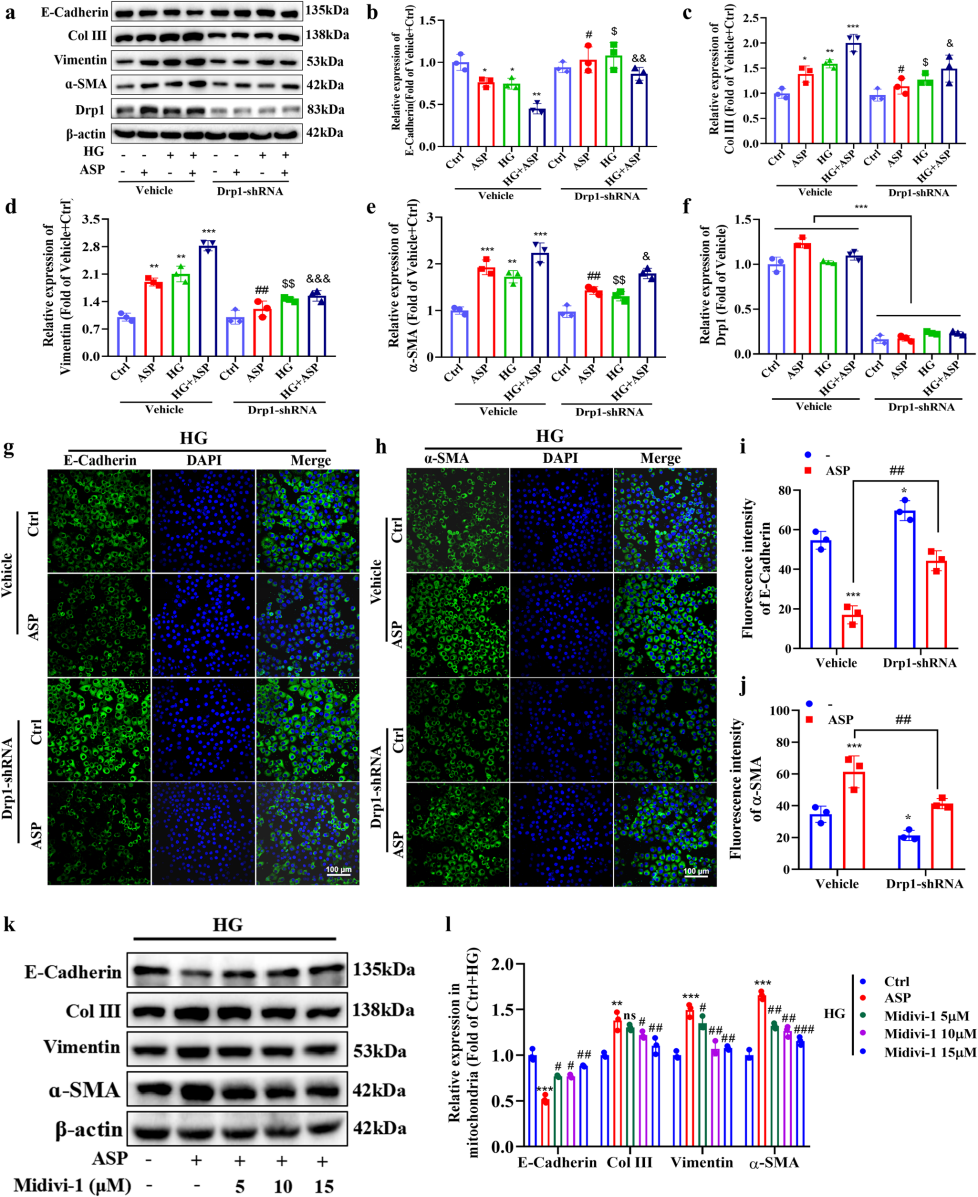
Pro‐injury and phenotypic transformation effects of ASP are mediated by Drp1. a–f) Representative Western blotting and quantitative data showing the expressions of E‐Cadherin, Collagen III (Col III), Vimentin, α‐SMA, and Drp1 in HK2 cells after Drp1 shRNA was transfected to the HK2 cells. β‐actin was used as a loading control (n = 3). g–j) Representative images of immunofluorescence staining and quantitative data of E‐Cadherin, α‐SMA in HK2 cells (n = 3). Scale bar: 100 µm. k, l) Representative Western blotting and quantitative data showing the expressions of E‐Cadherin, Collagen III (Col III), Vimentin, and α‐SMA in HK2 cells. β‐actin was used as a loading control (n = 3). Data were presented as mean ± SEM. Statistical analysis was performed using one‐way (l) or two‐way ANOVA (b‐f, i, j) followed by Tukey's multiple comparison test. All tests were two tailed. ^*^ *P* < 0.05, ^**^ *P* < 0.01, ^***^ *P*<0.001 versus Vehicle+Ctrl or HG+Ctrl; ^#^ *P* < 0.05, ^##^ *P* < 0.01, ^###^ *P* < 0.001 versus Vehicle+ASP or HG+ASP; ^$^ *P* < 0.05, ^$$^ *P* < 0.01 versus Vehicle+HG; ^&^ *P* < 0.05, ^&&^ *P* < 0.01,^&&&^ *P* < 0.001 versus Vehicle+HG+ASP.

Next, we investigated the underlying mechanisms by which ASP induces TEC injury and phenotypic transformation. Drp1 serves as an important protein regulating mitochondrial dynamics, and it remains uncertain whether the impaired effects of ASP on TEC would be mediated by Drp1 in DKD mice. To address it, we knocked down Drp1 (Figure S9a–c, Supporting Information) and utilized pharmacologic inhibitors of Drp1 in HK2 cells. Our results displayed that down‐regulation of Drp1 markedly antagonized the ASP‐mediated TEC injury and phenotypic transformation, especially under the HG, evidenced as increased E‐Cadherin, and decreased Vimentin, ɑ‐SMA as well as Col III (**Figure** **5**a–f). Immunofluorescence data also showed that Drp1 down‐regulation significantly reversed the decrease of E‐Cadherin (Figure 5g,i) and the increase of α‐SMA (Figure 5h,j) triggered by ASP. In addition, Midivi‐1, a Drp1 inhibitor, significantly reversed the TEC phenotypic transformation induced by ASP in HK2 cells, and the inhibitory effect of Midivi‐1 was concentration‐dependent (Figure 5k,l). These dates indicate that the pro‐injury and phenotypic transformation effects of ASP are mediated by Drp1.

### 2.6 ASP Intervention Enhances Drp1‐SUMO1 Modification

**Figure 6:**
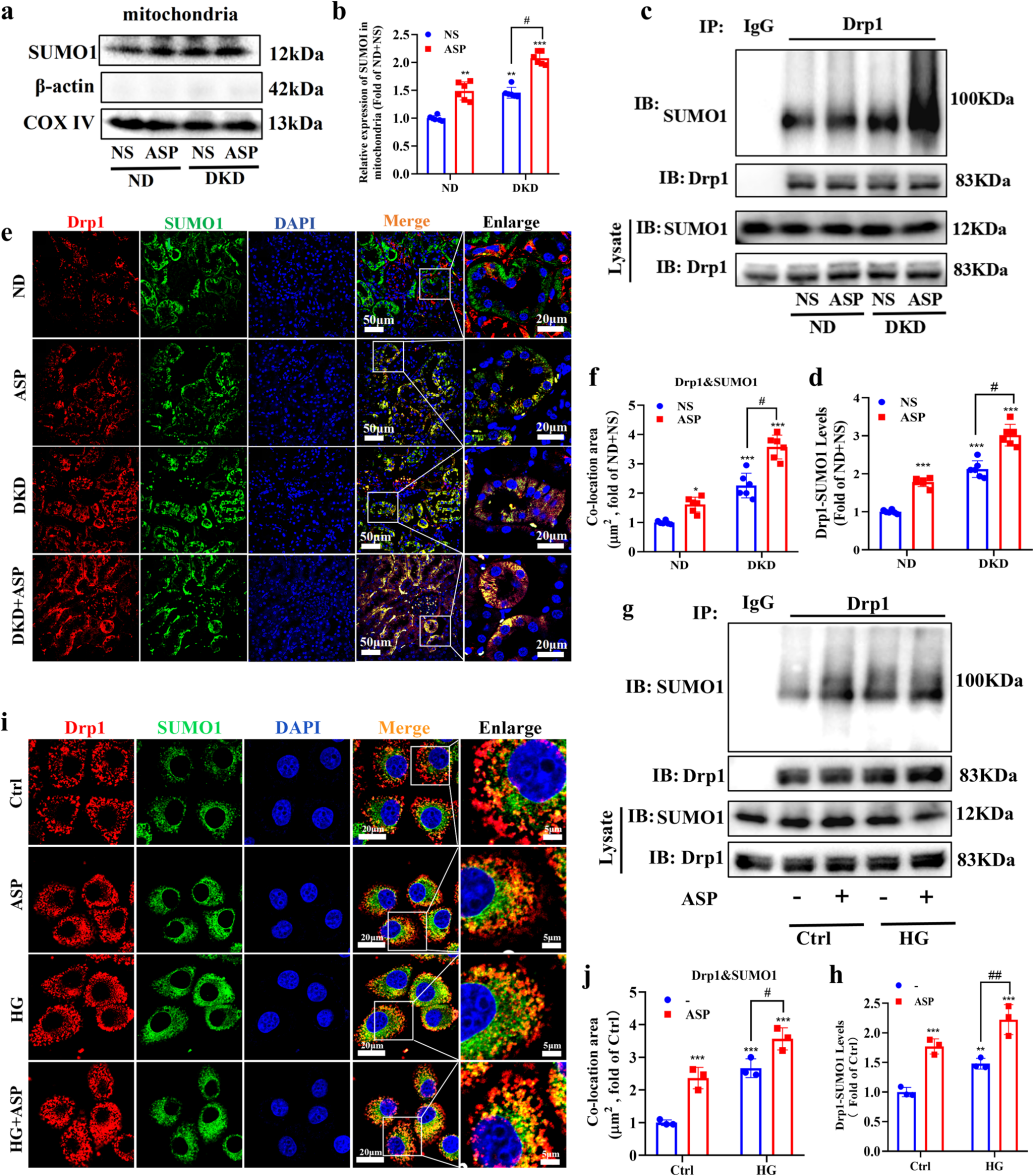
ASP intervention enhances Drp1‐SUMO1 modification. a, b) Representative Western blotting and quantitative data showing the expression of SUMO1 in the mitochondrial fraction in kidneys. COX IV was used as loading controls (n = 6). c, d) SUMOylation of Drp1 levels detected by Co‐IP in kidneys (n = 6). e, f) Co‐localization of Drp1 with SUMO1 and its quantitative data in kidneys (n = 6). Drp1 (red), SUMO1 (green), DAPI (blue). Scale bar: original images, 50 µm; enlarged images, 20 µm. g, h) SUMOylation of Drp1 levels detected by Co‐IP in HK2 cells (n = 3). i, j) Co‐localization of Drp1 with SUMO1 and its quantitative data in HK2 cells (n = 3). Drp1 (red), SUMO1 (green), DAPI (blue). Scale bar: original images, 20 µm; enlarged images, 5 µm. Data were presented as mean ± SEM. Statistical analysis was performed using two‐way ANOVA followed by Tukey's multiple comparison test (b, f, d, j, h). All tests were two tailed. ^*^ *P* < 0.05, ^**^ *P* < 0.01, ^***^ *P* < 0.001 versus ND+NS or Ctrl; ^#^ *P* < 0.05, ^##^ *P* < 0.01 versus DKD+NS or HG.

It is reported that Drp1 SUMOylation stabilizes its conformation and influences its localization and activity. Over‐modification of Drp1 by SUMO1 causes excessive accumulation of Drp1 in OMM, leading to abnormal mitochondrial fission.^[^ ^33^ ^]^ Our results exhibited that ASP intervention significantly elevated SUMO1 levels, which were consistent with the elevated levels of Drp1 in mitochondria. Furthermore, the combined intervention of ASP with STZ/HFD further elevated the levels of SUMO1 (**Figure** **6**a,b). Co‐IP results further demonstrated that ASP intervention, under the condition of whether ND or DKD markedly increased the levels of Drp1‐SUMO1 modification (Figure 6c,d), suggesting that ASP intervention promotes Drp1 SUMOylation. Besides, we investigated the in situ expressions and co‐localization of Drp1 and SUMO1 in the kidneys of DKD mice by immunofluorescence. The results revealed that ASP promoted the in situ expression and co‐localization of Drp1 and SUMO1 (Figure 6e,f). Similar results were obtained from the in vitro data (Figure 6g–j). Taken together, these data suggest that ASP intervention enhances Drp1 SUMOylation modification in vivo and in vitro. In addition, we examined the effects of ASP^−/−^ on the modification of Drp1‐SUMO1 in the kidneys of DKD mice. The results showed that ASP^−/−^ significantly reduced the levels of Drp1‐SUMO1 (Figure S10a,b, Supporting Information) and the co‐location of Drp1 and SUMO1 (Figure S10c,d, Supporting Information).

### 2.7 Drp1‐SUMOylation Site Mutation Alleviates TEC Mitochondrial Dysfunction and Phenotypic Transformation Induced by ASP

**Figure 7:**
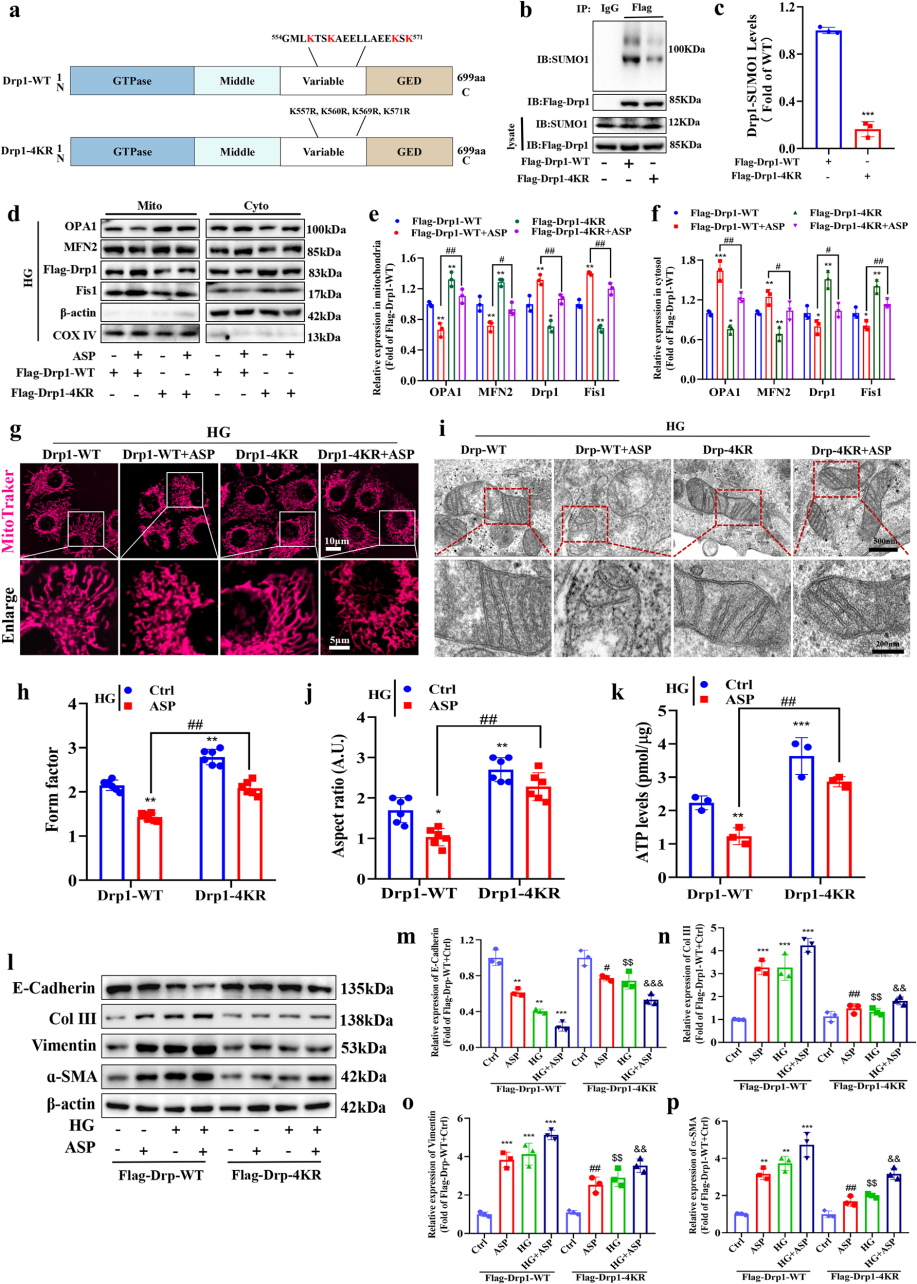
Drp1‐SUMOylation site mutation alleviates TEC mitochondrial dysfunction and phenotypic transformation induced by ASP. a) Domain structures of wild‐type Drp1 (Drp1‐WT) and a non‐SUMOylatable Drp1 mutant (Drp1‐4KR). A D‐octadecapeptide sequence, comprising 18 amino acid residues (554–571) encompassing in the Variable domain. Drp1‐4KR was created by substituting four lysine residues (K, red) which are SUMOylation sites in the D‐octadecapeptide with arginine residues (R). b, c) SUMOylation of Drp1 levels detected by Co‐IP of HK2 cells in Flag‐Drp1‐4KR groups (n = 3). d–f) Representative Western blotting and quantitative data showing the expressions of OPA1, MFN2, Flag‐Drp1 and Fis1 in cytosolic fraction and mitochondrial fraction of HK2 cells. β‐actin was used as a marker for the cytoplasmic fraction, and COX IV was used as a marker for the mitochondrial fraction (n = 3). g, h) Representative images of MitoTraker (red) and its quantitative data in HK2 cells (n = 3). Scale bar: original images, 10 µm; enlarged images, 5 µm. i, j) Representative images of TEM and quantitative data in HK2 cells (n = 3). Scale bar: original images, 500 nm; enlarged images, 200 nm. k) ATP levels in HK2 cells (n = 3). l–p) Representative Western blotting and quantitative data showing the expressions of E‐Cadherin, Collagen III (Col III), Vimentin, and α‐SMA in HK2 cells. β‐actin was used as a loading control (n = 3). Data were presented as mean ± SEM. Statistical analysis was performed using the unpaired Student's *t*‐test (c), one‐way (e, f) or two‐way ANOVA (h–k, m–p) followed by Tukey's multiple comparison test. All tests were two tailed. ^*^ *P* < 0.05, ^**^ *P* < 0.01, ^***^ *P* < 0.001 versus Drp1‐WT+Ctrl; ^#^ *P* < 0.05, ^##^ *P* < 0.01 versus Drp1‐WT+ASP; ^$$^ *P* < 0.01 versus Drp1‐WT+HG; ^&&^ *P* < 0.001 versus Drp1‐WT+HG+ASP.

It is reported that Drp1 contains GTPase domain, a Middle domain, a Variable domain, and GTPase‐effector domain (GED) and the SUMOylation of Drp1 (NM_0 05690.4) occurs at K557, K560, K569, and K571 sites in the Variable domain.^[^ ^34^ ^]^ To further elucidate the roles of Drp1 SUMOylation in TEC injury and phenotypic transformation induced by ASP, a mutation of Drp1 SUMOylation sites at K557, K560, K569, and K571, abbreviated as Drp1‐4KR was constructed (**Figure** **7**a). Our results showed that Drp1‐4KR mutant notably reduced the Drp1‐SUMO1 levels compared to Drp1‐WT (Figure 7b,c) and alleviated the mitochondrial dynamics disorder induced by ASP, mainly manifested as the increased expressions of mitochondrial fusion proteins OPA1 and MFN2 and decreased expressions of mitochondrial fission proteins Drp1 and Fis1 (Figure 7d–f) and mitochondrial fragmentation (Figure 7g–j). Moreover, Drp1‐4KR alleviated the impairment of mitochondrial function caused by ASP in HK2, mainly indicated as the elevated ATP levels (Figure 7k). Also, Drp1‐4KR significantly reversed the TEC phenotypic transformation induced by ASP, evidenced as the increased epithelial marker E‐Cadherin and the decreased mesenchymal markers Col III, Vimentin, and α‐SMA (Figure 7l–p). Overall, these data indicate that Drp1 SUMOylation site mutation mitigates the mitochondrial dysfunction and phenotypic transformation of TEC induced by ASP.

### 2.8 ASP Pro‐TEC Injury and Phenotypic Transformation Mediated by Drp1‐SUMO1 is Regulated by SENP1 and PIAS1

**Figure 8:**
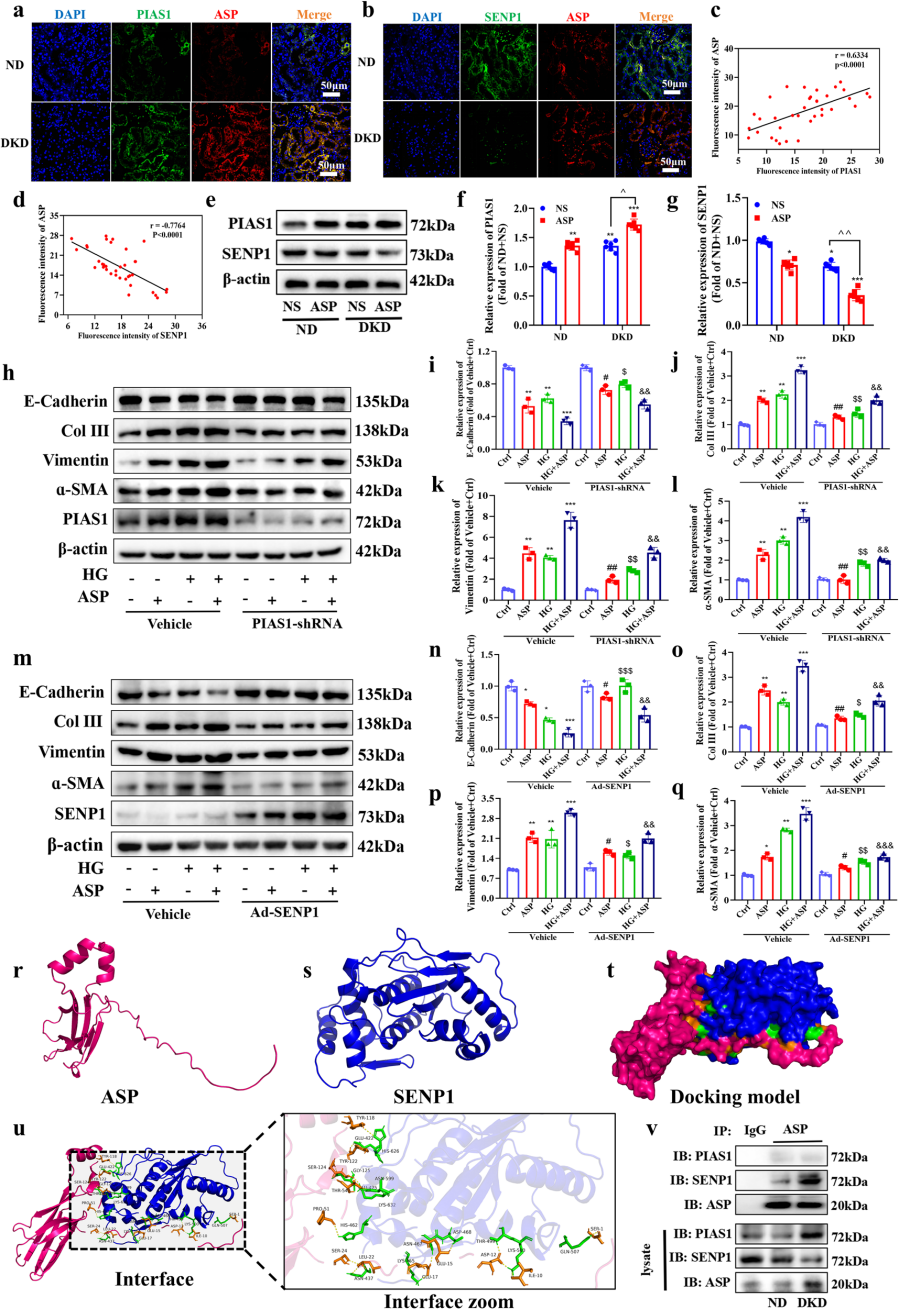
ASP pro‐TEC injury and phenotypic transformation mediated by Drp1‐SUMO1 is regulated by SENP1 and PIAS1. a) Representative images of immunofluorescence staining of ASP and PIAS1 in kidneys. ASP (red), PIAS1 (green), and DAPI (blue). Scale bar: 50 µm. n = 6. b) Representative images of immunofluorescence staining of ASP and SENP1 in kidneys. ASP (red), SENP1 (green), and DAPI (blue). Scale bar: 50 µm. c) Correlation between ASP and PIAS1 in DKD mice (n = 36). d) Correlation between the ASP and SENP1 in DKD mice (n = 36). e–g) Representative Western blotting and quantitative data showing the expression of PIAS1 and SENP1 in HK2 cells. β‐actin was used as a loading control. h–q) Representative Western blotting and quantitative data showing the expressions of E‐Cadherin, Collagen III (Col III), Vimentin, and α‐SMA of HK2 cells. β‐actin was used as a loading control. r) The 3D structure of ASP was predicted using the SWISS‐MODEL database. s) The 3D structures of SENP1 were obtained from the PDB database. t, u) Molecular docking demonstrated the interaction between ASP (red) and SENP1 (blue) (ΔiG = ‐12.5 kcal mol^−1^), which was obtained through the HADOCK molecular docking platform. v) Interaction between ASP and PIAS1 or SENP1 detected by Co‐IP in the kidneys of ND and DKD mice. Data were presented as mean ± SEM. Statistical analysis was performed using the two‐way ANOVA followed by Tukey's multiple comparison test (f, g, i‐l, n‐q). Pearson correlation was used to calculate the correlation coefficient (c, d). All tests were two tailed. ^*^ *P* < 0.05, ^**^ *P* < 0.01, ^***^ *P* < 0.001 versus ND+NS or Vehicle+Ctrl; ^*P* < 0.05, ^^*P* < 0.01 versus DKD+NS; ^#^ *P* < 0.05, ^##^ *P* < 0.01 versus Vehicle+ASP; ^$^ *P* < 0.05, ^$$^ *P* < 0.01, ^$$$^ *P* < 0.001 versus Vehicle+HG; ^&&^ *P* < 0.001 versus Vehicle+HG+ASP.

PIAS1 SUMOylates substrate proteins as E3 ligase of SUMOylation while SENP1 de‐SUMOylates them as a de‐SUMOylase. Therefore, they determine the substrate‐SUMO1 levels. As ASP promotes the TEC injury and phenotypic transformation by elevating Drp1‐SUMO1 levels, it remains unclear whether ASP would affect PIAS1 and SENP1. Hence, we detected the expressions of PIAS1, SENP1, and ASP in the kidneys of DKD mice. We found that the expressions of PIAS1 and ASP were elevated in a positive correlation manner (**Figure** **8**a,c), while the expressions of SENP1 and ASP embodied a negative correlation (Figure 8b,d). Similarly, ASP intervention notably increased the expression of PIAS1 and decreased the expression of SENP1 in the kidneys of DKD mice (Figure 8e–g). Subsequently, we performed the rescue experiments by PIAS1 knockdown and SENP1 over‐expression in HK2 cells. The results revealed that both PIAS1 knockdown (Figure S11a,b, Supporting Information) and SENP1 over‐expression (Figure S11c,d, Supporting Information) significantly reduced the Drp1‐SUMO1 levels. More importantly, both PIAS1 knockdown (Figure 8h–l) and SENP1 over‐expression (Figure 8m–q) reversed the phenotypic transformation induced by ASP in HK2 cells, characterized by increased E‐Cadherin and decreased Col III, Vimentin and α‐SMA, indicating that ASP promotes the TEC phenotypic transformation by up‐regulating the expression of PIAS1 and down‐regulating the expression of SENP1, and also suggesting that PIAS1 and SENP1 would be therapeutic targets of DKD. Subsequently, it was still unclear whether ASP would interact with PIAS1 or SENP1. Molecular docking results showed that ASP and SENP1 had a strong combined energy (ΔiG = −12.5 kcal mol^−1^, Figure 8r–u), which was further verified by Co‐IP results in the kidneys of DKD mice (Figure 8v). However, to our surprise, there was no physical interaction between ASP and PIAS1, suggesting that ASP affects PIAS1, maybe through other manners, which needs to be further explored.

### 2.9 Neutralization of ASP Alleviates TEC Injury and Phenotypic Transformation in DKD Mice

**Figure 9:**
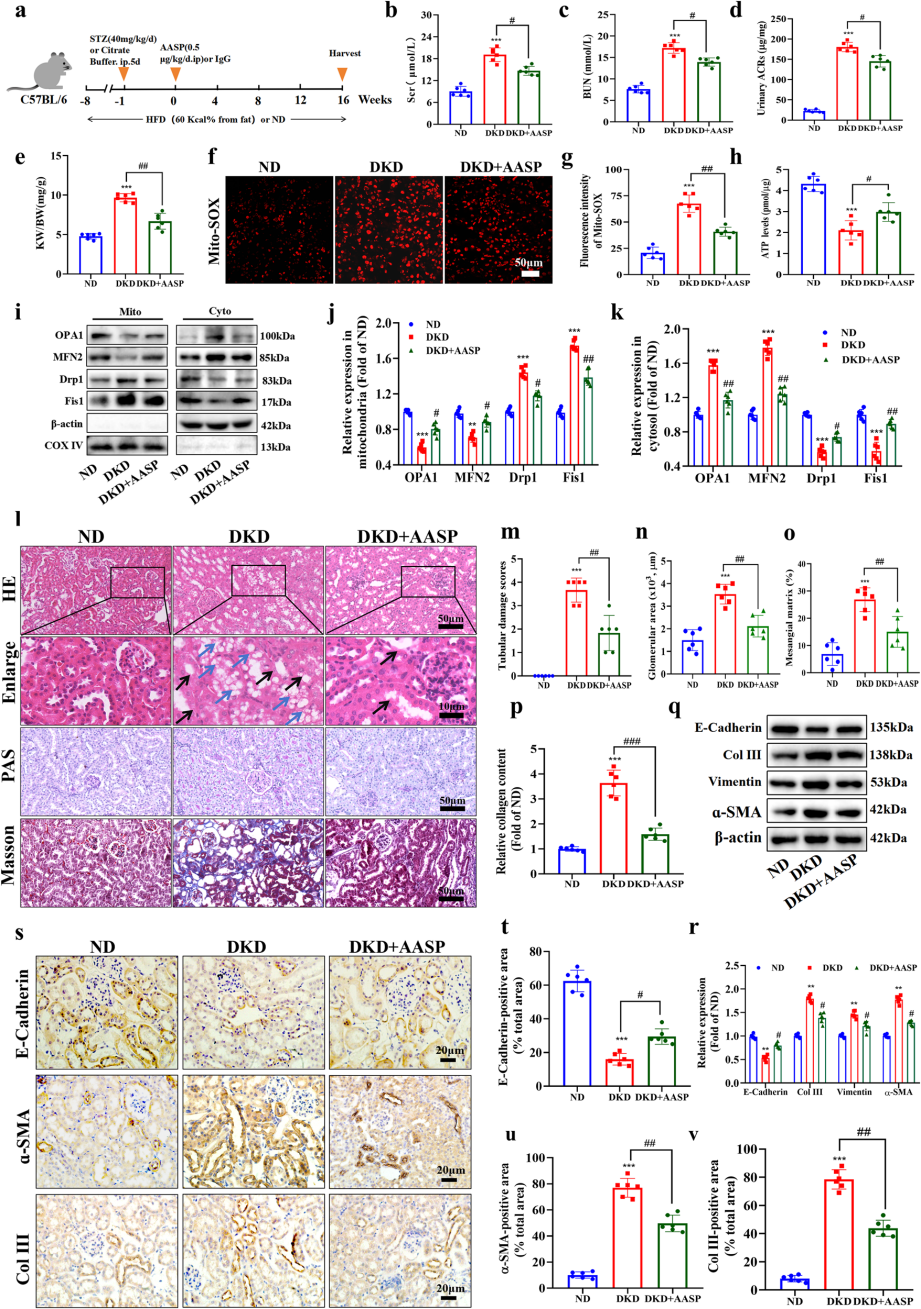
Neutralization of ASP alleviates TEC injury and phenotypic transformation in DKD mice. a) Schematic of the experimental design. STZ/HFD‐induced T2DM mice were treated with IgG or 0.5 µg · kg^−1^ · d^−1^ ASP neutralizing antibody (AASP) for 16 weeks. b, c) The levels of Scr and BUN levels in mice (n = 6). d, e) The urine albumin to creatinine ratios (Urinary ACRs) and kidney weight (KW)/body weight (BW) ratio in mice (n = 6). f, g) Representative images of MitoSOX (red) and its quantitative data in kidneys (n = 6). Scale bar: 50 µm. h) ATP levels in kidneys (n = 6). i–k) Representative Western blotting and quantitative data showing the expressions of OPA1, MFN2, Drp1 and Fis1 in cytosolic fraction and mitochondrial fraction of kidneys. β‐actin was used as a marker for the cytoplasmic fraction, and COX IV was used as a marker for the mitochondrial fraction (n = 6). l–p) Representative images and quantitative data of (l–n) hematoxylin and eosin (H&E, black arrow: swelling, blue arrow: vacuolation), (l, o) periodic acid–Schiff (PAS), and (l, p) Masson's trichrome staining in kidneys (n = 6). Scale bar: original images, 50 µm; enlarged images, 10 µm. q, r) Representative Western blotting and quantitative data showing the expressions of E‐Cadherin, Collagen III (Col III), Vimentin, and α‐SMA in kidneys. β‐actin was used as a loading control (n = 6). s–v) Representative images of immunohistochemical staining and quantitative data of E‐Cadherin, α‐SMA, and Collagen III (Col III) in kidneys (n = 6). Scale bar: 20 µm. Data were presented as mean ± SEM. Statistical analysis was performed using the one‐way ANOVA followed by Tukey's multiple comparison test in all panels except for (a), (f), (i), (l), (m), (q), and (s). The nonparametric Mann‐Whitney test was applied for statistical analysis of tubular injury scores (m). All tests were two tailed. ^**^ *P* < 0.01, ^***^ *P* < 0.001 versus ND; ^#^ *P* < 0.05, ^##^ *P* < 0.01, ^###^ *P* < 0.01 versus DKD.

The above studies suggest that ASP may be a promising target for the treatment of DKD. ASP is a newly discovered adipokine, and up to date there are no drugs that can combat ASP. Herein, an ASP neutralization antibody (AASP) was utilized to evaluate whether it can resist the negative effects of ASP on TEC. DKD mice induced by STZ/HFD were treated with AASP (**Figure** **9**a). We found that AASP treatment markedly reduced the levels of Scr, BUN, urinary ACRs and KW/BW ratio compared with DKD mice (Figure 9b–e). Further research showed that AASP significantly reduced the content of mitochondrial ROS (Figure 9f,g), increased the levels of ATP (Figure 9h), and improved the mitochondrial dynamics homeostasis (Figure 9i–k) in kidneys of DKD mice. These data indicate that AASP has protective effects on mitochondrial dynamics and function of kidneys in DKD mice. Moreover, H&E staining results displayed that AASP reduced TEC swelling (black arrow), vacuolation (blue arrow, Figure 9l,m), and glomerular hypertrophy (Figure 9l,n). Also, PAS and Masson staining data exhibited that AASP alleviated mesangial matrix expansion (Figure 9l,o) and collagen deposition (Figure 9l,p). The results of Western blotting (Figure 9q,r) and immunohistochemistry (Figure 9s–v) showed that AASP inhibited the progress of TEC phenotypic transformation, evidenced by increasing E‐Cadherin and decreasing the expressions of Col III, Vimentin, and α‐SMA. Altogether, these results demonstrate that AASP alleviates TEC injury and phenotypic transformation as well as mitochondrial dysfunction in DKD mice.

## 3 Discussion

DKD affects ≈40% of diabetic patients, and ≈20%–40% of those with DKD will develop to ESRD.^[^ ^35^ ^]^ Once renal structural damage occurs, it is exceedingly difficult to reverse the damage that has already taken place.^[^ ^36^, ^37^, ^38^ ^]^ TEC injury occurs in the early stage of ESRD and is regulated by hyperglycemia, transforming growth factor beta 1 (TGF‐β), interleukin 6 (IL‐6), and other factors.^[^ ^39^, ^40^ ^]^ Here, we found that the levels of circulating ASP were positively correlated with the levels of BUN and Scr, and the levels of urinal ASP were significantly elevated in the DKD mice. Therefore, we for the first time propose that ASP might be a novel renal injury factor.

As a newly discovered adipokine, ASP levels were found elevated in the kidneys in patients with DKD.^[^ ^29^ ^]^ The proximal tubule plays a key role in maintaining metabolic homeostasis in the body through the reabsorption of nutrients (glucose and amino acids). ASP is primarily involved in the regulation of glucose metabolism and energy homeostasis. Our present results also demonstrate that ASP is involved in the development of DKD by interacting with the TEC. Our results show that ASP is abundantly accumulated in the proximal tubules and seriously damages the structure and function of the renal tubules in DKD mice. It is reported that tubular damage initiates TEC phenotypic transformation, including epithelial marker loss and mesenchymal marker acquisition, primarily representing a maladaptive response to injury that accelerates fibrosis.^[^ ^35^ ^]^ We found that ASP decreased the expression of epithelial markers (E‐Cadherin), while increasing the expression of mesenchymal markers (Col III, Vimentin, and α‐SMA) in mouse kidneys and HK2 cells. More importantly, ASP intervention results in TEC mitochondrial dysfunction, which further causes glycolipid metabolic disorder and TEC damage. Our data have shown that ASP intervention exacerbates the glucose and lipid metabolic disorder in DKD mice, which is mainly attributed to TEC mitochondrial dysfunction induced by ASP. As previously reported, we constructed adipose tissue‐specific ASP‐deficient mice using CRISPR‐Cas9 technology.^[^ ^24^, ^25^, ^41^ ^]^ We found that ASP deficiency significantly reversed TEC phenotypic transformation and ameliorated the mitochondrial function. In addition, to explore whether ASP^−/−^ affects other adipokine levels, we tested the levels of serum leptin, adiponection, and FFA. Our results suggest that ASP^−/−^ may be combined with other adipokines to regulate the DKD process. Therefore, we conclude that ASP aggravates the TEC injury and phenotypic transformation in DKD mice, which deserves our further attention on its mechanisms.

Mitochondria are the primary organelles responsible for energy metabolism in the kidney regardless of whether the health or DKD status.^[^ ^7^, ^42^, ^43^ ^]^ Renal re‐absorption mainly occurs in the proximal tubule, which is enriched in mitochondria due to its high energy demand.^[^ ^44^, ^45^, ^46^ ^]^ Mitochondrial dynamics homeostasis is necessary for maintaining normal mitochondrial function. Our previous studies found that ASP disturbed the mitochondrial dynamics mediated by Drp1 and caused vascular endothelial cell dysfunction.^[^ ^25^ ^]^ In order to clarify whether the effects of ASP on TEC would be related to the mitochondrial dynamics, we focused on the regulatory roles of ASP on Drp1. We found that ASP promoted the aggregation of Drp1 on mitochondria and disrupted mitochondrial dynamics homeostasis, including the reduced expression of fusion proteins OPA1 and MFN2, increased expression of fission proteins Drp1 and Fis1, and elevated mitochondrial fragmentation in the kidneys of both normal and DKD mice. Similar results were obtained from the in vitro experiments. Mitochondrial dynamics disturbance often results in mitochondrial dysfunction. Our results revealed that ASP intervention also caused mitochondrial dysfunction, such as increased mitochondrial ROS levels, decreased ATP levels, and mitochondrial membrane potential (decreased JC‐1 aggregates). In contrast, ASP deficiency significantly reversed the impairments of mitochondrial dynamics and function by ASP.

Our results indicate that Drp1 plays an important role in the process of mitochondrial dynamics disorder induced by ASP. As expected, both Drp1 knockdown and intervention with Midivi‐1 (a Drp1 inhibitor) in HK2 cells significantly reversed the disruptive effects of ASP on mitochondrial dynamics and TEC injury and phenotypic transformation. It is reported that post‐translational modification of Drp1 affects its translocation from the cytosol to the mitochondria and activity. It is well established that Drp1 SUMOylation can greatly stabilize Drp1 conformation and inhibit its degradation by lysosomes.^[^ ^14^, ^47^ ^]^ Our study showed that Drp1‐SUMO1 modification was enhanced in the kidneys of DKD mice, and ASP intervention further enhanced the levels of Drp1‐SUMO1. On the contrary, we mutated the SUMO modification sites of Drp1 and found that the disturbance of mitochondrial dynamics and TEC injury, and phenotypic transformation induced by ASP in HK2 cells were greatly abolished. Altogether, these results suggest that Drp1‐SUMO1 over‐modification plays an important role in the process of TEC injury and phenotypic transformation induced by ASP.

PIAS1 and SENP1 are E3‐ubiquitin ligase and SUMO‐specific protease, respectively, mediating protein SUMO modification and de‐SUMO modification.^[^ ^48^ ^]^ Regulation of PIAS1 and SENP1 determines the SUMO modification levels of substrate proteins. Our data showed that PIAS1 and SENP1 also mediated Drp1‐SUMO1 modification in DKD. To our surprise, the expression level of ASP was positively correlated with that of PIAS1, but negatively correlated with that of SENP1. In addition, we found that both PIAS1 knockdown and SENP1 over‐expression reversed the damaged effects of ASP on HK2 cells. However, the molecular docking results showed that ASP can interact with SENP1, and the results of Co‐IP further verified the interaction between ASP and SENP1 rather than PIAS1. Therefore, it is rational that PIAS1 and SENP1 would be potential therapeutic targets for treating DKD. Excitingly, we found that AASP, an antibody to ASP, mitigated the TEC injury and phenotypic transformation, rescued the mitochondrial dynamics homeostasis, reduced mitochondrial ROSand increased ATP levels, suggesting that the negative regulation for ASP would be a therapeutic strategy for DKD.

However, there are still some limitations and unsolved issues. First, this study primarily focused on the injury effects of ASP on renal tubules, while paying less attention to glomeruli. Second, we only investigated the effects of Drp1‐SUMO1 modification on DKD induced by ASP, and did not confirm which SUMO modification site could play a major role in this process. Finally, although several studies have shown that the receptors of ASP in the central nervous system and liver are olfr734^[^ ^49^ ^]^ and Ptprd,^[^ ^50^ ^]^ respectively, what ASP receptor would work and whether the interaction between ASP and SENP1 would be mediated through membrane receptors of TEC need to be further investigated. Addressing these issues is of great significance for suppressing the negative effects of ASP on TEC.

In conclusion, we found that ASP intervention damages TEC and mitochondrial dynamic homeostasis in DKD mice and HK2 cells. In contrast, either ASP deficiency or AASP significantly alleviates the TEC injury and phenotypic transformation in DKD mice. Mechanistically, ASP causes and even aggravates TEC injury and phenotypic transformation by promoting excessive Drp1‐SUMO1 modification, in which PIAS1 and SENP1 are highly correlated with ASP and play important regulatory roles (Figure 10). The findings indicate that ASP would be an early marker of DKD and an important target of DKD treatment.

## 4 Experimental Section

### Experimental Animals

Animal (Nanfang Model Biotechnology, Shanghai, CHN) experiments were approved by the Laboratory Animal Ethic and Welfare Committee of Nanchang University (approval No. NCULAE‐20241213001) and conducted in accordance with the National Research Council Guide for the Care and Use of Laboratory Animals (8th Edition, 2011, Washington, DC published by the National Academies Press). The animals were housed in standard cages under controlled conditions of temperature (23 ± 1 °C) and humidity (50–60%), with a 12‐h light/dark cycle, and had ad libitum access to water. Adipose tissue‐specific ASP‐deficient mice were generated as previously described.^[^ ^24^, ^25^ ^]^ Random allocation of groups was conducted using a computer‐based random order generator.

### Mouse Type 2 Diabetes Mellitus (T2DM) Models

Mouse T2DM model was established as previously described by Xie, et al.^[^ ^51^ ^]^ Briefly, after 7 weeks of high‐fat diet (HFD) feeding, ASP^−/−^ mice and ASP^fl/fl^ littermates were injected intraperitoneally with STZ (40 mg · kg^−1^ body weight in citrate buffer, pH 4.5, Sigma–Aldrich, St. Louis, MO, USA, #18883‐66‐4) for 5 days to induce hyperglycemia. The mice were then given 10% sucrose for 24 h to prevent hypoglycemic shock. One week after the injection, mice with fasting plasma glucose (FPG) ≥11.1 mmol L^−1^ were selected for the following experiments. After continuous HFD‐feeding for 16 weeks, FPG, serum creatinine (Scr), blood urea nitrogen (BUN), kidney weight/body weight ratios (KW/BW), and urine albumin to creatinine ratios (urinary ACRs) were detected. When the above indicators far exceeded the normal values, the DKD model was successfully established.

### Protocols In Vivo

For the experiment of ASP (QYAOBIO, Hubei, CHN) intervention in DKD mice, twenty‐four 8‐week‐old C57BL/6 male mice were randomly allocated to four groups (six per group): normal diet (ND), ASP, DKD and DKD+ASP groups. After the model of T2DM was established by STZ/HFD, the mice in the ASP and DKD+ASP groups were intraperitoneally injected with ASP (0.5 µg · kg^−1^· d^−1^, ip) for 16 weeks. The mice in the ND and DKD groups were injected with saline daily. Meanwhile, the mice in the ND and ASP groups were fed with ND and those in the DKD and DKD+ASP groups were fed with HFD (60/100 kcal fat (soybean oil, lard), 20/100 kcal carbohydrate (500 kcal maltodextrin, 275 kcal sucrose) and 20/100 kcal protein, (Xietong, Inc., Nanjing, CHN) during the whole experiment, respectively.

For the experiment of the effects of ASP^−/−^ on DKD mice, twelve 8‐week‐old C57BL/6 male ASP^fl/fl^ mice were randomly divided into ND and DKD groups (six mice per group). In addition, twelve 8‐week‐old C57BL/6 male ASP^−/−^ C57BL/6 mice were randomly divided into ND and DKD groups (six mice per group). After the model of T2DM was established by STZ/HFD, the mice in ND (ASP^fl/fl^+ND and ASP^−/−^+ND) groups and DKD (ASP^fl/fl^+DKD and ASP^−/−^+DKD) groups were fed with ND and HFD for 16 weeks, respectively.

For the experiment of the effects of AASP (a neutralization antibody to ASP, for more information about AASP, please refer to our previously published paper^[^ ^25^ ^]^), eighteen 8‐week‐old C57BL/6 male mice were randomly allocated to three groups (six mice per group), namely, ND, DKD, and DKD+AASP groups. After the model of T2DM was established by STZ/HFD, the mice in the DKD+AASP group were injected with AASP (0.5 µg · kg^−1^· d^−1^, ip), and those in the ND and DKD groups were injected with IgG (a control of AASP, ip) daily. The mice in the ND group were fed with ND, and the mice in the DKD (DKD, DKD+AASP) groups were fed with HFD during the whole experiment, respectively. Blood and urine samples of all 3 batches of mice were respectively collected every two weeks for the detection of the relevant indicators. The mice were sacrificed at the end of the experiments by inhaling 3% (v/v) isoflurane, and kidney and liver tissues were collected for further examinations.

### Cell Culture

Human renal tubular epithelial cell line (HK‐2 cells, ATCC, USA) was propagated in Dulbecco's Modified Eagle Medium/Nutrient Mixture F‐12 (DMEM/F‐12) medium (Gibco Life Technologies, Carlsbad, CA, USA), supplemented with 10% fetal bovine serum (FBS, Tianhang, Zhejiang, CHN, #13011‐8611) and 1% penicillin/streptomycin (Gibco Life Technologies, Carlsbad, CA, USA). WT‐3T3‐L1 preadipocytes (ATCC, USA) and ASP‐deficient 3T3‐L1 preadipocytes (ASP^−/−^, the construction method of ASP‐deficient cells and the methods of inducing 3T3‐L1 maturation can be referred to our previous paper^[^ ^24^ ^]^) were conventionally cultured in H‐DMEM (Solarbio, Beijing, CHN) supplemented with 10% FBS (Tianhang, Zhejiang, CHN, #13011‐8611) and 1% penicillin/streptomycin (Gibco Life Technologies, Carlsbad, CA, USA). All cells were cultured in a humidified atmosphere containing 5% CO_2_ at 37 °C. The cells were undergone distinct treatments, as specified in the Figure Legends.

### Statistical analysis

Data were presented as mean±SEM. Unpaired Student's *t* test (2‐sided) was used for statistical analysis between two groups. Nonparametric Mann‐Whitney test was applied for statistical analysis of tubular injury scores. One‐way or two‐way ANOVA followed by Tukey's multiple comparison test was used for multiple group comparisons. Pearson correlation analysis was used to calculate the correlation coefficient. *P* < 0.05 was considered statistically significant. GraphPad Prism 8.0 (GraphPad Software) was used for statistical analysis. Detailed methods were provided in the Figure Legends and Supporting Information section.

## Conflict of Interest

The authors declare no conflict of interest.

## Author Contributions

Q.Q.H., Q.R.H., and G.Z. conceived and designed the study. Q.Q.H. and X.X. assisted in the experimental designs. Q.Q.H., X.X., S.C., and Y.W. conducted the experiments and acquired the data. L.W., W.L., and C.L. analyzed the data. Q.Q.H. and Q.R.H. drafted and revised the manuscript. All authors approved the final version of the manuscript.

## Supporting information



Supporting Information

## Data Availability

The data that support the findings of this study are available from the corresponding author upon reasonable request.
