# Supplementary material for: Asprosin Aggravates Tubular Epithelial Cell Injury and Phenotypic Transformation via Mitochondrial Dynamics Disorder Mediated by Excessive Drp1 SUMOylation in Diabetic Nephropathy Mice
Source: Adv Sci (Weinh). 2025 Aug 27;12(39):e03259. doi: 10.1002/advs.202503259 (PMC12533405; doi:10.1002/advs.202503259)
Supplement: Supplementary file 1 — Supporting Information [file ADVS-12-e03259-s001.pdf]

ADVANCED SCIENCE
Open Access

Supporting Information

for *Adv. Sci.*, DOI 10.1002/advs.202503259

Asprosin Aggravates Tubular Epithelial Cell Injury and Phenotypic Transformation via Mitochondrial Dynamics Disorder Mediated by Excessive Drp1 SUMOylation in Diabetic Nephropathy Mice

Qianqian Huang, Xiaowei Xiong, Sheng Chen, Yuan Wang, Li Wang, Wentao Liu, Chen Liu, Guohua Zeng and Qiren Huang**

Supplementary Materials

METHODS

Evaluation of renal function

Serum creatinine (Scr) were assessed using creatinine assay kit (Jiancheng, Nanjing, CHN, #C011-2-1). Blood urea nitrogen (BUN) were assessed by urea assay kit (Jiancheng, Nanjing, CHN, #C013-2-1) and urine albuminwere assessed by albumin assay kit (Jiancheng, Nanjing, CHN, #A028-2-1). Experimental process references to manufacturer's protocol. The samples were detected by a microplate reader (PerkinElmer, Shanghai, CHN).

Production of anti-ASP polyclonal antibodies (AASP)

The anti-ASP polyclonal antibody (AASP) was custom-synthesized by China Peptides Co., Ltd. (Shanghai, China) through the following standardized protocol:

1) Antigen conjugation: target peptides were covalently linked to carrier proteins using keyhole limpet hemocyanin (KLH) as the immunogen and bovine serum albumin (BSA) for subsequent assay validation.
2) Immunization protocol: pre-immunization serum samples were obtained from two rabbits prior to initiating a four-round immunization regimen to ensure baseline reference data.
3) Serum monitoring: post-immunization sera were iteratively collected and systematically evaluated for antibody responsiveness.
4) Titer quantification: antibody titers were determined via ELISA, followed by terminal blood collection and antiserum isolation through centrifugation.

5) Antibody purification: final affinity purification was performed using Protein A chromatography to obtain IgG fractions with enhanced specificity.

Transmission electron microscopy (TEM)

Mitochondrial ultra-structure was detected by TEM according to the previous reports.[1, 2] HK2 cells were fixed in 2.5% glutaraldehyde with 0.1M sodium cacodylate (pH 7.4) for 72 h at 4 °C. Samples were further incubated with 2% osmium tetroxide and 0.1 M sodium cacodylate (pH 7.4) for 1 h at room temperature. Ultrathin sections were stained with lead citrate and uranyl acetate and viewed on a microscope (HT7800 Hitachi, Tokyo, Japan). Briefly, negatives were digitized, and images with a final magnitude of up to X3,000 were obtained. Twenty mitochondria were analyzed per section in a blinded fashion, under X3000 magnification (n=3, per group). Dysmorphic mitochondria were defined as the mitochondria with a focal loss of visible cristae, clustering of residual cristae at the peripheral mitochondrial membrane, and fragmented. Subsequent analysis of mitochondrial morphology parameters was performed using the Image J software (NIH, USA).

GTT and ITT

GTT and ITT were performed as described in previous reports.[3] Briefly, mice were fasted for 8 h before GTT and ITT. Subsequently, mice were anesthetized with 3% (v/v) isoflurane, and blood samples were collected from the tail vein to record baseline glucose levels at 0 min. After that, glucose solution (2 g/kg) or insulin (1 U/kg) was injected intraperitoneally. Plasma glucose levels were then measured at 15, 30, 60, 90, 120, 180, and 150 min of post-injection. The time-concentration curve was

drawn, and the area under the curve (AUC) was calculated.

Detection of plasmic glucose, serum T-CHO and TG

The plasmic levels (mmol/L) of fasting glucose in mice was measured by glucose oxidase method using a kit (Abbkine, Wuhan, CHN, #KTB1300). Serum levels of T-CHO (mmol/L) and TG (mmol/L) were measured using corresponding kits (Jiancheng, Nanjing, China). Following the manufacturer's instructions, measurements were performed at the designated optical density (OD) on a microplate reader (PerkinElmer, Shanghai, CHN).

Blood pressure measurement in mice

Systolic blood pressure (SBP) was measured with the Blood Pressure System (Kent Scientific Corporation, NH, USA) using the mouse tail cuff method. Briefly, the mouse tail was tied to the tail cuff, and the mice was placed in the restraint at 37°C. After a week of adaptive training, the mice were conditioned to the experimental environment. Subsequently, 10 measurements were taken at every 1-minute interval for each mouse, and the values were averaged.

Adipokine detection

The levels of ASP (Camilo, Nanjing, CHN, #2M-KMLJM221034m), leptin (Solarbio, Beijing, CHN, #SEKM-0105) and adiponectin (Beyotime, Shanghai, CHN, #PA002) in serum were determined using ELISA kits according to the kit instructions. Free fatty acids (FFA) were determined using assay kit (Solarbio, Beijing, CHN, #BC0595). The samples were detected by a microplate reader (PerkinElmer, Shanghai, CHN).

Kidney histology and tubular injury scoring

Kidney tissues were fixed with 4% paraformaldehyde for 24 h and then embedded in paraffin. Tissue sections (5 μm) were used for hematoxylin and eosin (H&E, Solarbio, Beijing, CHN, #G1120) staining and periodic acid-Schiff (PAS, Solarbio, Beijing, CHN, #G1281) staining. The stainings were carried out according to the previous researches.[2, 4] H&E staining was used to analyze glomerular areas and PAS staining was used to evaluate mesangial matrix index. Assessment of the glomerular was performed by pixel counts at a minimum of 20 glomeruli per section in a blinded fashion, under 40X magnification.

The results of H&E and PAS staining were combined to score the degree of renal tubular injury. Tubular injury was scored in the cortical region on a scale from 0-4, based on tubular dilatation, vacuolation and tubular structure.[5] The tubules were evaluated according to the following scoring system: 0 = normal tubules, 1 = less than 25% tubules injured, 2 = 25%-50% tubules injured, 3 = 50%-75% tubules injured and 4 = more than 75% tubules injured. Scoring was performed at minimum of 10 cortical fields (X40 original magnification) per section per mouse and averaged (n = 6 mice per group). Nonparametric Mann-Whitney test was applied for statistical analysis of tubular injury scores. Representative images were acquired with microscope (Olympus, JPN) and analyzed with Image J software (NIH, USA).

Masson Trichrome Staining

Masson Trichrome (Solarbio, Beijing, CHN, #G1340) staining were performed to evaluate the tubulointerstitial collagen deposition area. Tissue sections (5 μm) were used for staining according to the manufacturer's instructions. The area of collagen

deposition was quantified at least 10 cortical fields (X400 original magnification) per Masson-stained kidney section per mouse (n = 6) and averaged. Representative images were acquired with microscope (Olympus, JPN) and analyzed with Image J software (NIH, USA).

Immunohistochemical staining

Immunohistochemical staining was performed as previously described.[6] Briefly, kidneys were fixed with 4% paraformaldehyde for 24 h and then 5 μm sections were cut from paraffin-embedded kidney tissues. The dewaxed slices were hydrated for antigen recovery and incubated with 3 % H_2O_2 and then heated in 0.1 mol/L sodium citrate (pH 6.0) at 95°C to 100°C for 15min for antigen retrieval. The slices were blocked with 5% bovine serum albumin (BSA, Boster, Wuhan, CHN, #AR0004) for 40min before incubation of primary antibody. Next, the slices were incubated with primary antibody at 4°C overnight. The list of primary antibodies is as follows: anti-E-Cadherin (1：200， Zhengneng，Chengdu，CHN，#340341) , anti-Col III (1:200, Zhengneng, Chengdu, CHN, #R23957) and anti-α-SMA (1:200, Affinity, Jiangsu, CHN, #AF1032). The next day, the slides were incubated with biotinylated goat anti-rabbit secondary antibody (1:200; Songon Biotech, Shanghai, CHN, #D110073) for 40 min at room temperature. Meanwhile, the primary antibody was replaced by non-immune IgG as negative control group. Finally, The color was developed with 3,3'-diaminobenzidine (DAB, Solarbio, Beijing, CHN, #DA1010). Representative images were acquired with microscope (Olympus, JPN) and quantified at least 10 cortical fields (X400 original magnification) per stained kidney section per mouse (n

= 6) and averaged and analyzed with Image J software ((NIH, USA).

Isolation of sub-cellular fractions

The cytosolic and mitochondrial fractions were isolated using the mitochondrial isolation kit (Solarbio, Beijing, CHN, #EX1320), according to the manufacturer's instructions.

Mitochondrial membrane potential determination (MMP)

MMP was measured using the JC-1 assay kit (Abbkine, Wuhan, CHN, #KTA4001), and the experiment was carried out according to the manufacturer's instructions. Representative images were acquired with a laser confocal microscope (Nikon, JPN) and analyzed with Image J software (NIH, USA).

Mitochondrial ROS measurement

Mitochondrial ROS was measured using a MitoSOX kit (Thermo Fisher Scientific, Waltham, MA, USA, #M36007).[7] Briefly, kidney cryosections or HK2 cells were incubated with MitoSOX for 10 min at 37°C. The stained cells or sections were then washed with phosphate-buffered saline and observed using a confocal laser scanning biological microscope (Nikon, JPN) or flow cytometer (Beckman Coulter, USA). Representative images were acquired with a laser confocal microscope (Nikon, JPN) and analyzed with Image J software (NIH, USA).

ATP measurement

ATP concentrations in kidneys and HK2 cells were measured using an ATP Fluorometric Assay Kit (Solarbio, Beijing, CHN, #BC0300), according to manufacturer’s protocol.

Immunofluorescence staining

The pretreatment of sections was consistent with immunohistochemistry. Immunofluorescence staining was performed as previously described.[8] The slices were disposed with Triton X-100 and blocked with 5 % BSA (Boster, Wuhan, CHN, #AR0004) for 40 min. Then, samples were incubated at 4°C overnight with the primary antibodies, followed by incubation with iFluor™ 594 conjugated goat anti-mouse IgG (HUABIO, Hangzhou, CHN, #HA1126) and iFluor™ 488 conjugated goat anti-rabbit IgG (HUABIO, Hangzhou, CHN, #HA1121). The primary antibodies in the study are as follows: anti-AQP1(1:200, Wanlei, Shenyang, CHN, WLH3886), anti-NCC (1:200, Abcam, Cambridge, MA, USA, #ab95302), anti-ASP (1:200, AdipoGen, Liestal, Switzerland, #Q61554), anti-Drp1 (1:200, Proteintech Biotech, Wuhan, CHN, #12957-1-AP), anti-Tomm20 (1:200, Abway, Shanghai, CHN, #CY5527) and anti-SUMO1 (1:200, HUABIO, Zhejiang, CHN, #ET1606-53). Samples were counterstained with 4', 6-diamidino-2-phenylindole, dihydrochloride (DAPI, Boster Biotech, Wuhan, CHN, #AR1176). The method of cell staining was consistent with that of tissue section. Representative images were acquired with a laser confocal microscope (Nikon, JPN) and analyzed with Image J software (NIH, USA).

Western blots

The kidney tissues or cells were lysed in RIPA lysis buffer (Keygen BioTECH, Jiangsu, CHN, #KGB5203-100) on ice, followed by centrifugation at 12,000 rpm for 15 min at 4°C. Next, the supernatant was collected and the protein concentration was

determined by BCA kit (SEVEN, Beijing, CHN, #SW101). The 30 μg protein was then isolated by SDS-PAGE and transferred to PVDF membrane (Millipore Corporation, Billerica, MA, USA). Subsequently, the samples were blocked with 7% nonfat milk for 2 h. After that, samples were incubated at 4 °C overnight with the primary antibodies, followed by staining with HRP-conjugated secondary antibodies (1:2000, Boster Biotech, Wuhan, CHN, BA1050, BA1054, respectively). Finally, the bands were visualized by the enhanced chemical luminescence solution under the Gel Imaging System (Bio-Rad Laboratories, CA, USA) and subsequently analyzed by the Image J software (NIH). The primary antibodies in the study are as follows: anti-E-Cadherin (1: 1000, Zhengneng, Chengdu, CHN, #340341), anti-Col III (1:1000, Zhengneng, Chengdu, CHN, #R23957), anti-Vimentin (1:1000, HUABIO, Zhejiang, CHN, #ET1610-39), anti-α-SMA (1:1000, Affinity, Jiangsu, CHN, #AF1032), anti-β-actin (1:1000, BOSTER, Hubei, CHN, #BA2305), anti-COX-IV (1:1000, HUABIO, Zhejiang, CHN, #RT1158), anti-OPA1 (HUABIO, Zhejiang, CHN, #ET1705-9), anti-MFN2 (Proteintech Biotech, Wuhan, CHN, #12186-1-AP), anti-Drp1 (1:1000, Proteintech Biotech, Wuhan, CHN, #12957-1-AP), anti-p-Drp1 ser616 (1:1000, Affinity, Shanghai, CHN, #AF8470), anti-Fis1 (1:1000, Zhengneng, Chengdu, CHN, #R26001), anti-SUMO1 (1:1000, HUABIO, Zhejiang, CHN, #ET1606-53), anti-SENP1 (1:1000, CST, Danvers, MA, USA, #11929), anti-PIAS1 (1:1000, CST, Danvers, MA, USA, #3550S), anti-ASP (1: 1000, AdipoGen, Liestal, Switzerland, #Q61554).

Co-immunoprecipitation assay (Co-IP)

Cells and tissues were lysed in RIPA lysis buffer (Keygen BioTECH, Jiangsu, CHN, #KGB5202-100) consisting of 50 mM Tris-HCl (pH of 7.4), 150 mM NaCl, 1% Triton X-100, 1% sodium deoxycholate and 0.1% SDS, which was supplemented with phenylmethylsulfonylfluoride (PMSF, MedChemExpress, NJ, USA, #HY-B0496), Na3VO4, DL-dithiothreitol (DTT) and 20 mM N-ethylmaleimide (NEM). Extracts from tissues or cells were incubated with the primary antibody overnight with a gentle shake on a shaker at 4°C. Subsequently, the samples were incubated with the Protein A/G magnetic beads (MedChemExpress, NJ, USA, #HY-K0202) on a roller at room temperature for 2-3 h. Eventually, the samples were detected by Western blotting as described previously.[9] The primary antibodies in the study are as follows: anti-Drp1(1:1000, Proteintech Biotech, Wuhan, CHN, #12957-1-AP), anti-SUMO1(1:1000, HUABIO, Zhejiang, CHN, #ET1606-53), anti-SENP1 (1:1000, CST, Danvers, MA, USA, #11929), anti-PIAS1(1:1000, CST, Danvers, MA,USA, #3550S), anti-ASP (1:1000, AdipoGen, Liestal, Switzerland, #Q61554) and anti-Flag (1:1000, MedChemExpress, NJ, USA, #HY-P80111).

Wound healing assay

HK2 cells (3×10^5) were seeded in 6-well plates and grown to a full confluence in DMEM/F12 supplemented with 10% FBS and 1% penicillin/streptomycin. The cells were scratched using a pipette tip and washed with PBS (MedChemExpress, NJ, USA, #HY-K3005) 3 times to remove the detached cells. The cells were then cultured with DMEM containing 30 mM glucose for 24 h. Wound healing was calculated according to the following formula: migration area (%) = (W0-WN)/W0 $\times$ 100%, where W0 is

the initial area of the wound and WN is the area of the wound at the time of measurement.

Infiltration assay

WT-3T3-L1 preadipocytes or ASP$^{-/-}$-3T3-L1 preadipocytes (1×10^4) were seeded and induced into mature adipocytes in 24-well plates. Next, HK2 cells were inoculated in the upper compartment of Transwell (8μm pore size, SAINING, Suzhou, CHN, #1102130). After upper compartment's cell adhesion, the cells were stimulated with 30 mM glucose for 24 h. Then, the upper compartment was placed in 24-well plates containing WT-3T3-L1 or ASP$^{-/-}$-3T3-L1. Thus, the HK2 cells and 3T3-L1 cells were co-cultured in DMEM with 10% FBS for 48 h. Finally, the infiltrated cells on the lower surface were stained with 0.5% crystal violet for 15 min. The cell infiltration was observed under Olympus IX71 Fluorescence Microscopy (Olympus, Japan).

Virus infection

Adenoviruses carrying PIAS1-siRNA (Ad-shPIAS1), adenoviruses carrying SENP1-mRNA (Ad-SENP1) and lentivirus carrying Drp1-shRNA were purchased from Genechem (Shanghai, CHN). Lentivirus carrying flag-Drp1-WT and flag-Drp-4KR (K557R, K560R, K569R and K571R) were purchased from Life-iLab (Shanghai, CHN).[9, 10] The process of virus transfection of HK2 cells was as follows: cells were first plated to achieve confluence, then exposed to a viral suspension at a specified multiplicity of infection (MOI), followed by incubation for virus adsorption. Afterward, the inoculum was replaced with fresh medium to allow for virus replication and gene expression, with infection progress monitored at set time points

to evaluate the infection efficiency and cellular response.

Molecular docking

Molecular docking of ASP with SENP1 was performed to understand their binding and interaction patterns. Based on the known ASP amino acid sequence, we used SWISS-MODEL (https://swissmodel.expasy.org/) automatic modeling server to construct the three-dimensional (3D) structure model. Besides, 3D structure of SENP1 (PDB ID: 2IYC) was obtained from the Protein Data Bank (https://www.rcsb.org/). For docking, the default parameters were used as described in the HADOCK (https://rascar.science.uu.nl/haddock2.4/) server. The docking results were analyzed visually using PyMOL 1.7.6 software. (www.pymol.org).

RNA-sequence

The date of RNA-sequence were examined by Magigene (Guangzhou, CHN). Weishengxin software (https://www.bioinformatics.com.cn/) was used for data analysis.

Graphical abstract

The graphical abstract was drawn by Figdraw (https://www.figdraw.com/).

FIGURES AND LEGENDS

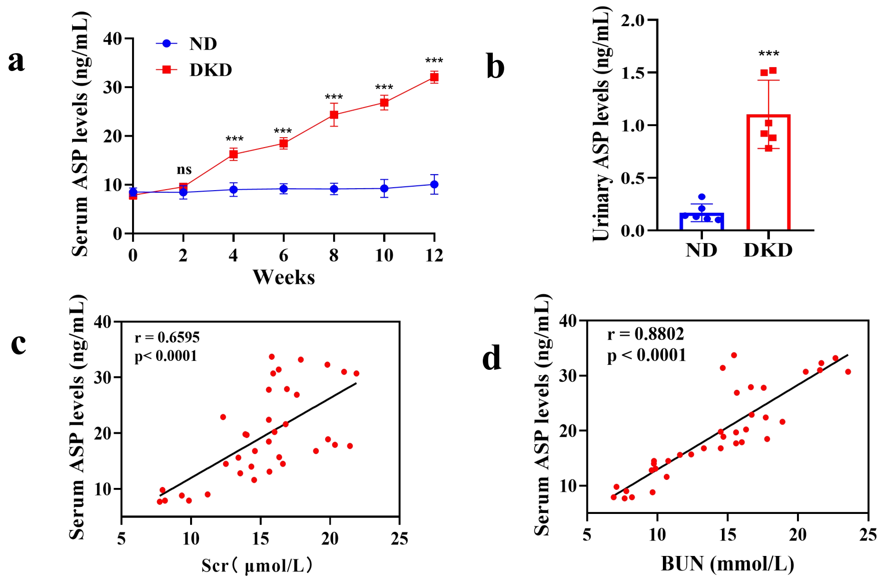

Figure S1. ASP is closely related to the development of DKD. (a) Serum ASP levels at various weeks in the mice of ND and DKD groups (n=6). (b) Urinary ASP levels at week 16 in the mice of ND and DKD groups (n=6). (c, d) The correlation between the circulating levels of ASP and the concentrations of serum creatinine (Scr) and blood urea nitrogen (BUN) in DKD mice (n=36). Data were presented as mean±SEM. Unpaired Student's t test (2-sided) was used in (a) and (b). Pearson correlation was used to calculate the correlation coefficient in (c) and (d). All tests were two tailed. ***P<0.001 versus ND.

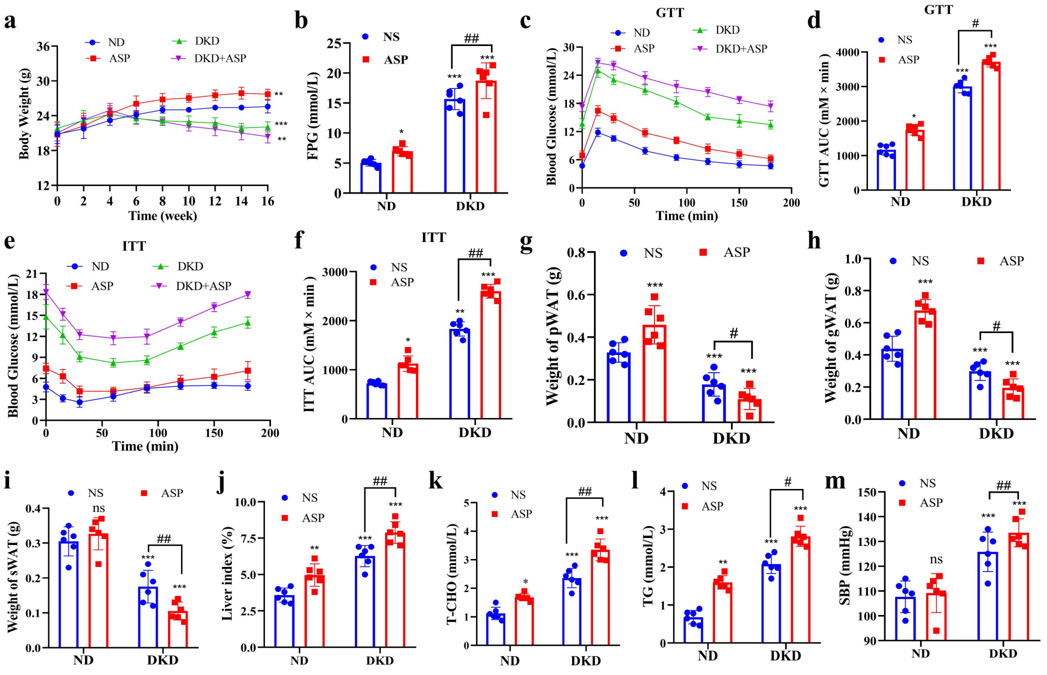

Figure S2. ASP disturbs glucose and lipid metabolism homeostasis in mice. (a, b) Body weight and fasting plasmic glucose (FPG) were detected. (c, d) Glucose tolerance test (GTT) curve were prepared and area under curve (AUC) were calculated. (e, f) Insulin tolerance test (ITT) curve were prepared and AUC were calculated. (g-i) Wet weights of perirenal white adipose tissue (pWAT), gonadal white adipose tissue (gWAT) and subcutaneous white adipose tissue (sWAT) were detected. (j) Liver index was detected. (k, l) Circulatory total Cholesterol (T-CHO) and triglyceride (TG) levels were detected. (m) Systolic blood pressures (SBP) was detected. Data were presented as mean ± SEM (n=6). Statistical comparison was performed using the two-way ANOVA followed by Tukey's multiple comparison test in (a-m). All tests were two tailed. **P*<0.05, ***P*<0.01, ****P*<0.001 versus ND+NS; #*P*<0.05, ##*P*<0.01 versus DKD+NS. ns, no signifcant difference versus ND+NS.

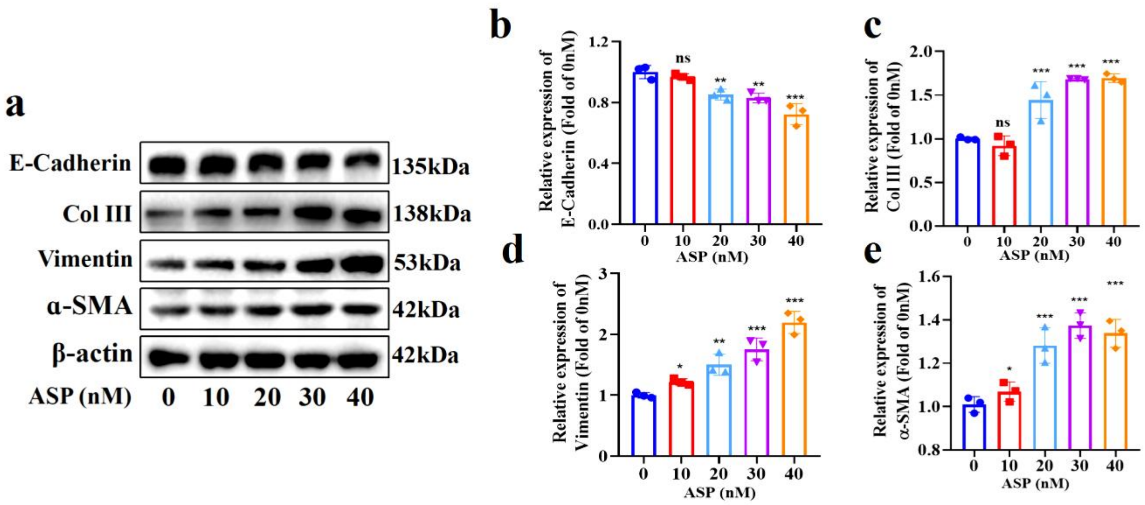

Figure S3. ASP promotes phenotypic transformation of HK2 in a concentration-dependent manner *in vitro*. (a-e) Western blotting and quantitative data showing the expressions of E-Cadherin, Collagen III (Col III), Vimentin and α-SMA under the conditions of gradient ASP concentration in HK2 cells. β-actin was used as loading controls (n=3). Data were presented as mean±SEM. Statistical comparison was performed using one-way ANOVA followed by Tukey's multiple comparison test in (e). All tests were two tailed. **P*<0.05, ***P*<0.01, ****P*<0.001 versus 0 nM. ns, no significant difference versus 0 nM.

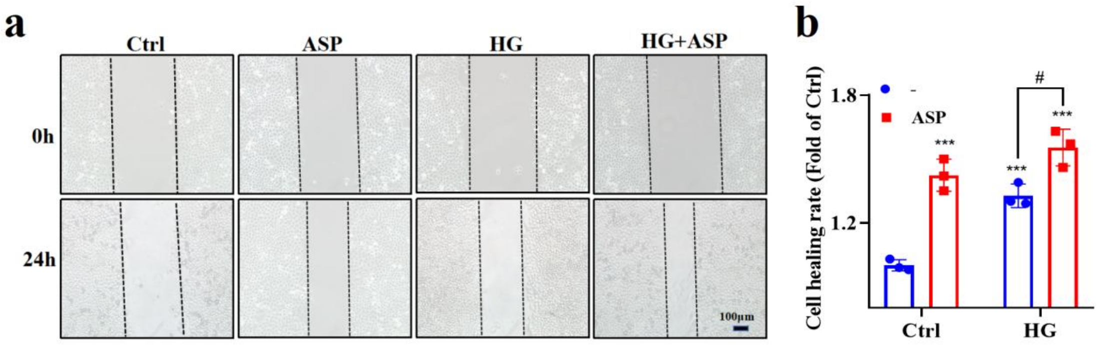

Figure S4. ASP promotes the migration of HK2 cells. (a, b) Representative images and quantification of the HK2 cells migration by the wound healing assay (n=3). Scale bar: 100 μm. Data were presented as mean±SEM. Statistical comparison was performed using the two-way ANOVA followed by Tukey's multiple comparison test in (b). All tests were two tailed. ****P*<0.001 versus Ctrl; #*P*<0.05 versus HG.

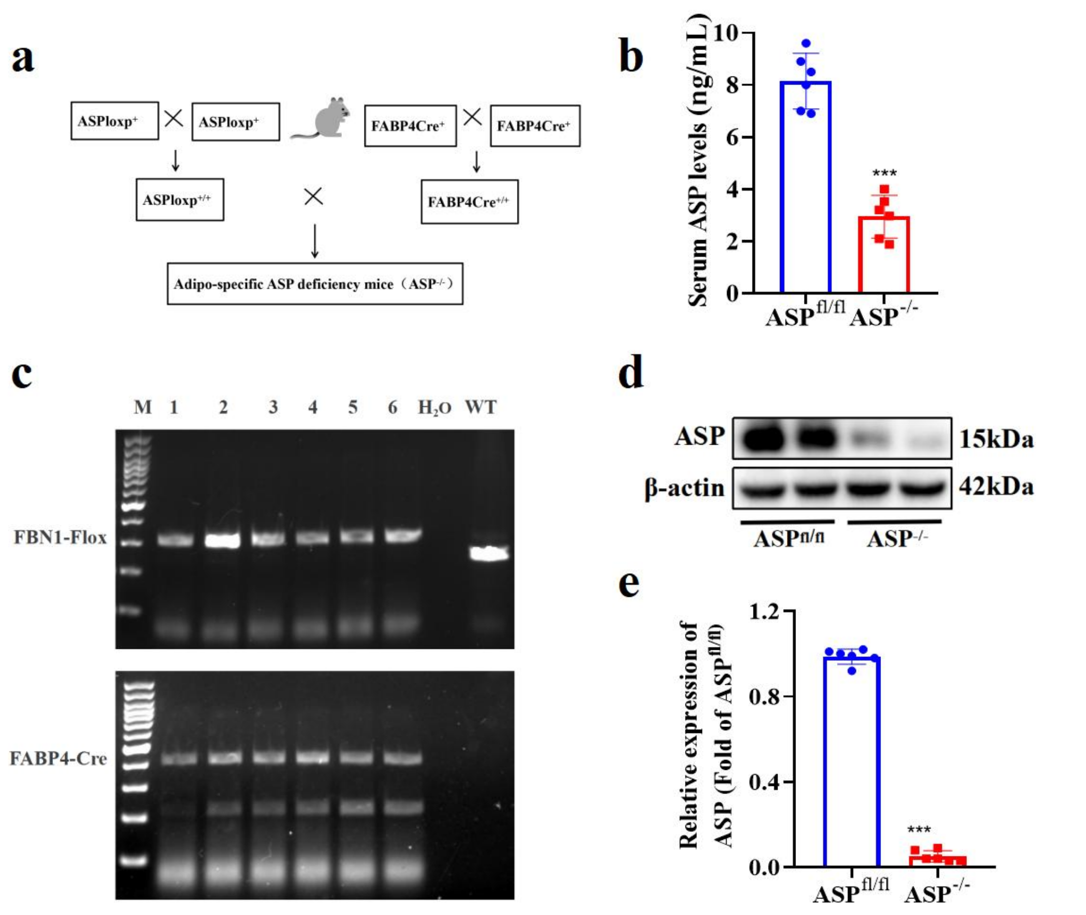

Figure S5. Establishment and identification of adipose tissue-specific ASP-deficient mice. (a) Schematic diagram of breeding mice with specific deficiency of ASP in white adipose tissue. (b) Serum ASP levels was detected by ELISA in $ASP^{fl/fl}$ and $ASP^{-/-}$mice. (c) Verification of $ASP^{-/-}$mice. (d, e) Western blotting verifying the ASP protein expression levels in white adipose tissue of $ASP^{fl/fl}$ and $ASP^{-/-}$ mice. Data were presented as mean±SEM (n=12). Unpaired Student's t test (2-sided) was used in (b) and (e). ***P<0.001 versus $ASP^{fl/fl}$.

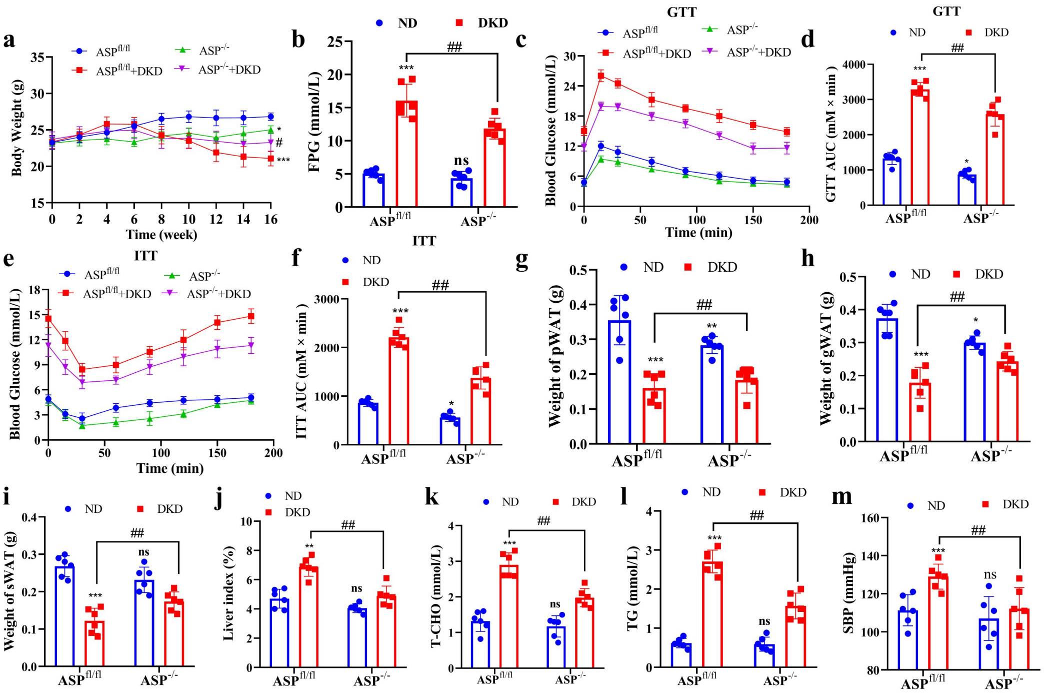

Figure S6. ASP deficiency ameliorates the glucose and lipid metabolic disorders in DKD mice. (a, b) Body weight, fasting plasmic glucose (FPG) were detected. (c, d) Glucose tolerance test (GTT) curve were measured and area under curve (AUC) were calculated. (e, f) Insulin tolerance test (ITT) curve were measured and AUC were calculated. (g-i) Wet weights of perirenal white adipose tissue (pWAT), gonadal white adipose tissue (gWAT) and subcutaneous white adipose tissue (sWAT) were detected. (j) Liver index was detected. (k, l) Circulatory total Cholesterol (T-CHO) and triglyceride (TG) levels were detected. (M) Systolic blood pressures (SBP) was detected. Data were presented as mean ± SEM (n=6). Statistical analysis was performed using the two-way ANOVA followed by Tukey's multiple comparison test in (a-m). All tests were two tailed. *$P<0.05$, **$P<0.01$, ***$P<0.001$ versus ASP$^{fl/fl}$+ND; $^{\#}P<0.05$, $^{\#\#}P<0.01$ versus ASP$^{fl/fl}$+DKD. ns, no signifcant difference versus ASP$^{fl/fl}$+ND.

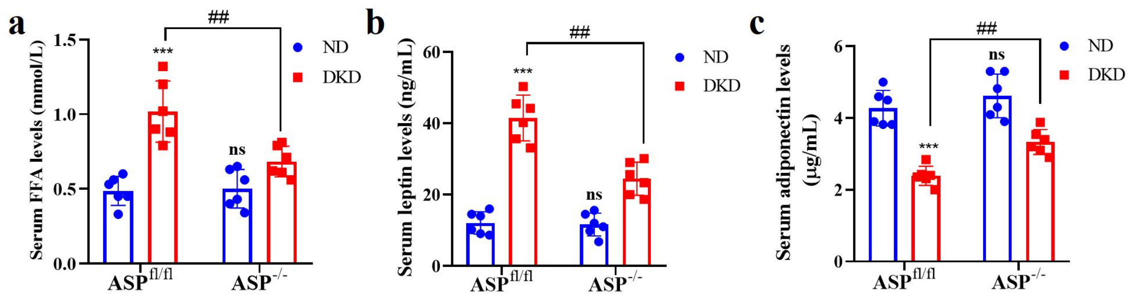

Figure S7. Effects of ASP $^{-/-}$ on serum FFA and adipokines in DKD mice. (a-c) Serum levels of free fatty acid (FFA), leptin and adiponectin were detected. Data were presented as mean ± SEM (n=6). Statistical analysis was performed using the two-way ANOVA followed by Tukey's multiple comparison test in (a-c). All tests were two tailed. ****P*<0.001 versus ASP$^{fl/fl}$+ND; $^{\#\#}$*P*<0.01 versus ASP$^{fl/fl}$+DKD. ns, no signifcant difference versus ASP$^{fl/fl}$+ND.

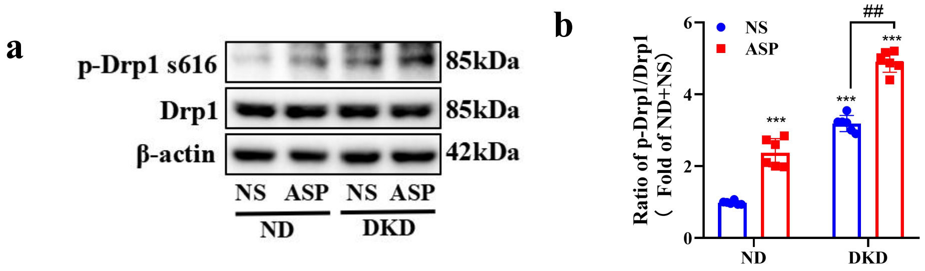

Figure S8. ASP increases the phosphorylation levels of Drp1 at s616. (a, b) Western blotting and quantitative analysis were performed with renal tissue of mice. β-actin was used as loading controls. Data were presented as mean ± SEM (n=6). Statistical analysis was performed using the two-way ANOVA followed by Tukey's multiple comparison test in (b). All tests were two tailed. ****P*<0.001 versus ND+NS; ##*P*<0.01 versus DKD+NS.

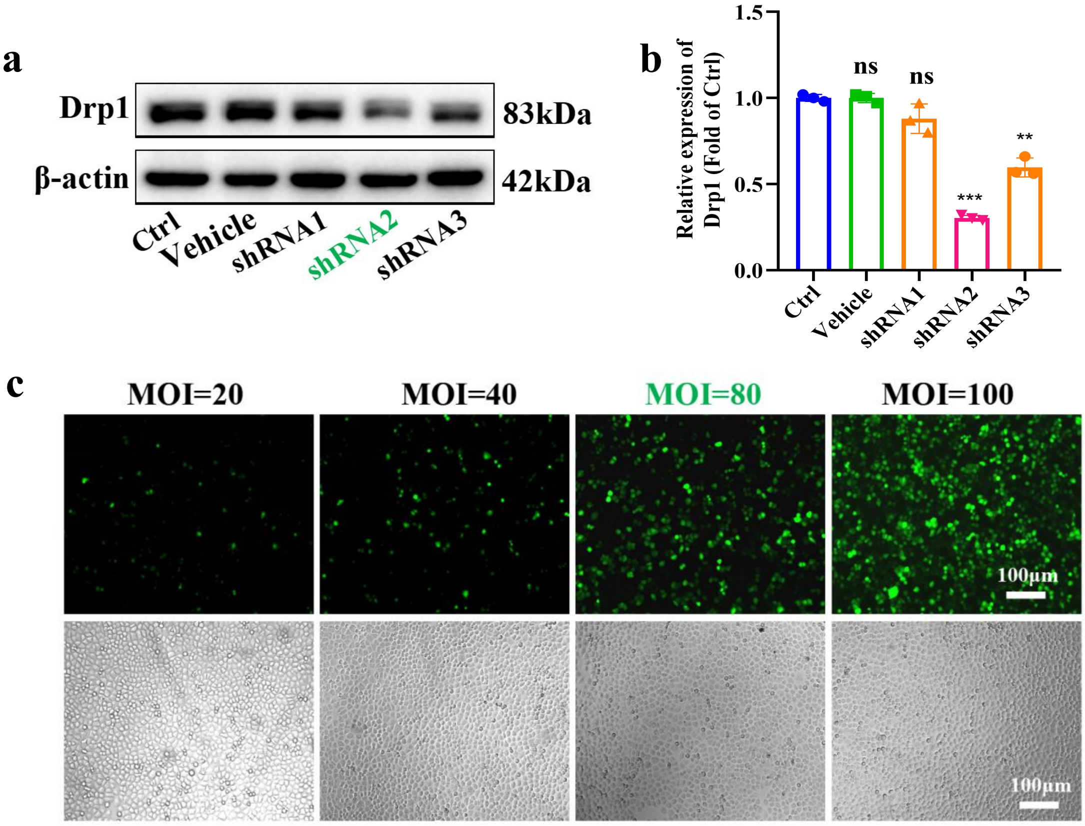

Figure S9. Identification of Drp1 down-regulation. (a, b) Western blotting and quantitative analysis were performed with HK2 cells. β-actin was used as loading controls.(c) Representative images of immunofluorescence staining of GFP-Drp1. Scale bar: 100 μm. Data were presented as mean±SEM (n=3). Statistical analysis was performed using one-way ANOVA followed by Tukey's multiple comparison test in (b). All tests were two tailed. ***P*<0.01, ****P*<0.001 versus Ctrl or Vehicle. ns, no signifcant difference, versus Ctrl or Vehicle.

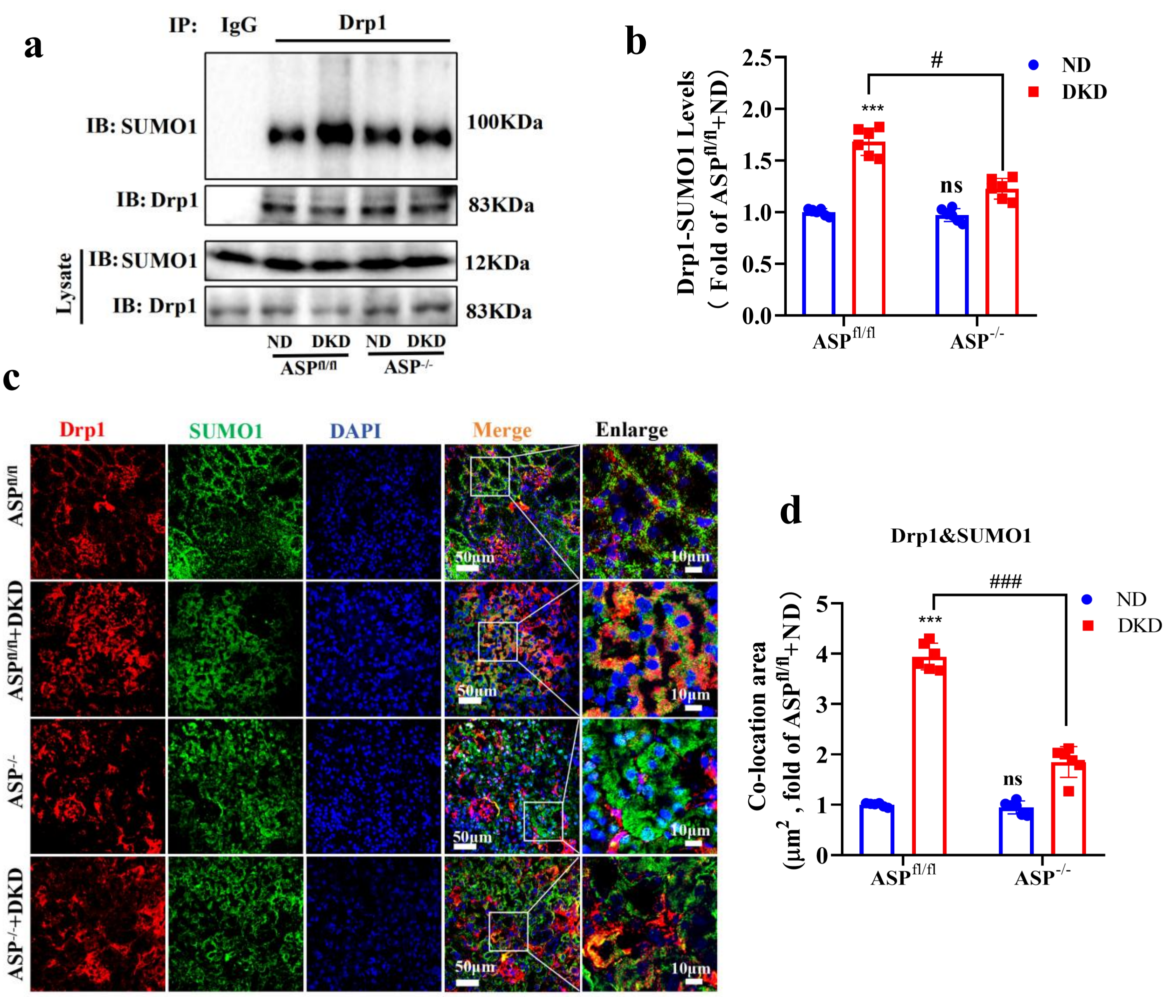

Figure S10. ASP$^{-/-}$ significantly reduces the levels of Drp1-SUMO1. (a, b) The levels of Drp1 SUMOylation were detected by Co-IP in kidneys. (c, d) Co-localization of Drp1 with SUMO1 and quantitative analysis were performed with mouse renal tissue. Drp1 (red), SUMO1 (green), DAPI (blue). Scale bar: original images, 50 μm; enlarged images, 10 μm. Data were presented as mean±SEM (n=6). Statistical analysis was performed using two-way ANOVA followed by Tukey's multiple comparison test (b, d). All tests were two tailed. $^{***}P<0.001$ versus ASP$^{fl/fl}$+ND; $^{\#}P<0.05$, $^{\#\#\#}P<0.001$ versus ASP$^{fl/fl}$+DKD. ns, no signifcant difference versus ASP$^{fl/fl}$+ND.

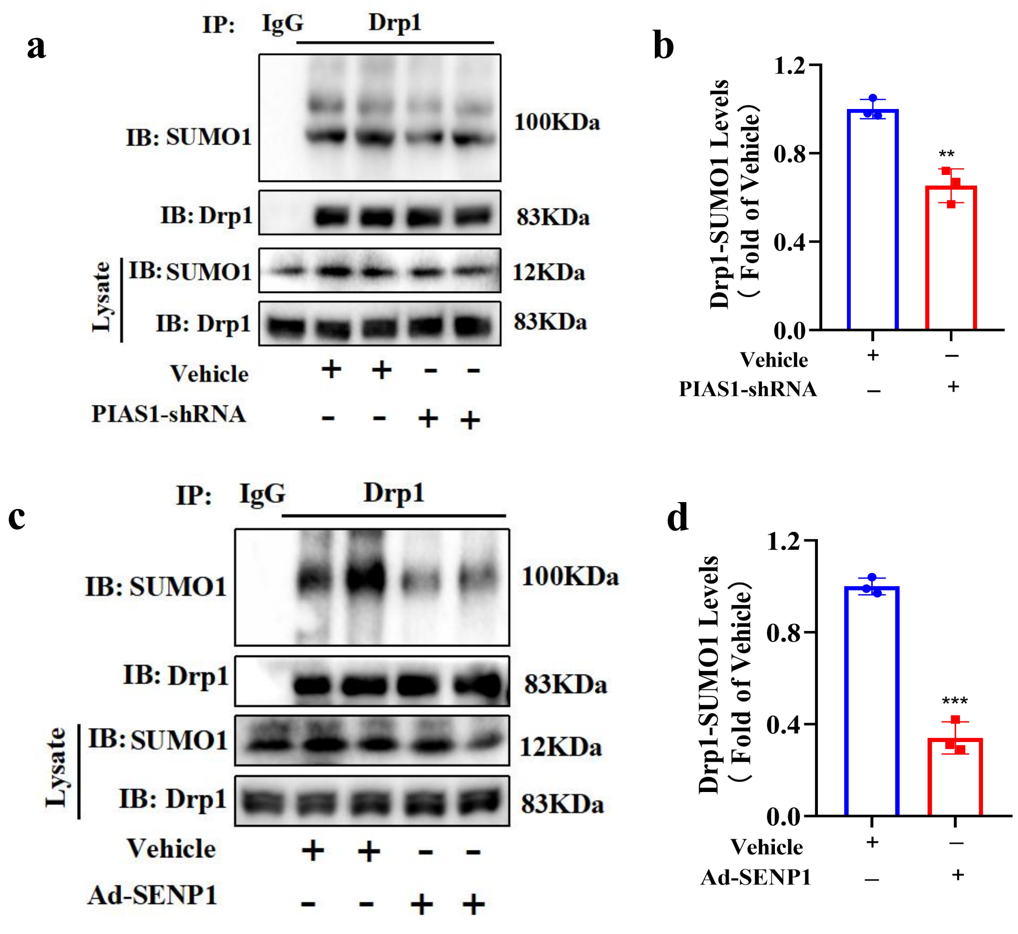

Figure S11. PIAS1 knockdown or SENP1 over-expression significantly reduces the Drp1-SUMO1 levels. (a-d) The levels of Drp1 SUMOylation were detected by Co-IP in HK2 cells. Data were presented as mean ± SEM. Statistical analysis was performed using the unpaired Student's t-test in (b) and (d). All tests were two tailed. ***P*<0.01, ****P*<0.001 versus Vehicle.

Supplementary references

[1] Yang K, Zheng Q, Luo M, Zhang R, Zhao S, Kang L, Lei X, Dong W. SIRT1 and its SUMOylation attenuate hyperoxia-induced lung injury by improving mitochondrial biogenesis and fusion. *Free Radic Biol Med* 2025; **236:** 98-115.

[2] Xie Y, E J, Cai H, Zhong F, Xiao W, Gordon RE, Wang L, Zheng YL, Zhang A, Lee K, He JC. Reticulon-1A mediates diabetic kidney disease progression through endoplasmic reticulum-mitochondrial contacts in tubular epithelial cells. *Kidney Int* 2022; **102:** 293-306.

[3] Zhang Y, Huang Q, Xiong X, Yin T, Chen S, Yuan W, Zeng G, Huang Q. Acacetin alleviates energy metabolism disorder through promoting white fat browning mediated by AC-cAMP pathway. *J Physiol Biochem* 2023; **79:** 529-541.

[4] Li Y, Hu Q, Li C, Liang K, Xiang Y, Hsiao H, Nguyen TK, Park PK, Egranov SD, Ambati CR, Putluri N, Hawke DH, Han L, Hung MC, Danesh FR, Yang L, Lin C. PTEN-induced partial epithelial-mesenchymal transition drives diabetic kidney disease. *J Clin Invest* 2019; **129:** 1129-1151.

[5] Li J, Sun X, Yang N, Ni J, Xie H, Guo H, Wang X, Zhou L, Liu J, Chen S, Wang X, Zhang Y, Yu C, Zhang W, Lu L. Phosphoglycerate mutase 5 initiates inflammation in acute kidney injury by triggering mitochondrial DNA release by dephosphorylating the pro-apoptotic protein Bax. *Kidney Int* 2023; **103:** 115-133.

[6] Jiang M, Wei Q, Dong G, Komatsu M, Su Y, Dong Z. Autophagy in proximal

tubules protects against acute kidney injury. *Kidney Int* 2012; **82:** 1271-1283.

[7] Qiao Y, Hu T, Yang B, Li H, Chen T, Yin D, He H, He M. Capsaicin alleviates the deteriorative mitochondrial function by upregulating 14-3-3eta in anoxic or anoxic/reoxygenated cardiomyocytes. *Oxid Med Cell Longev* 2020; **2020:** 1750289.

[8] Li H, Zhao X, Zheng L, Wang X, Lin S, Shen J, Ren H, Li Y, Qiu Q, Wang Z. Bruceine A protects against diabetic kidney disease via inhibiting galectin-1. *Kidney International* 2022; **102:** 521-535.

[9] Huang J, Xie P, Dong Y, An W. Inhibition of Drp1 SUMOylation by ALR protects the liver from ischemia-reperfusion injury. *Cell Death Differ* 2021; **28:** 1174-1192.

[10] Yamada S, Sato A, Ishihara N, Akiyama H, Sakakibara SI. Drp1 SUMO/deSUMOylation by Senp5 isoforms influences ER tubulation and mitochondrial dynamics to regulate brain development. *iScience* 2021; **24:** 103484.
